# Landscape of heart proteome changes in a diet-induced obesity model

**DOI:** 10.1038/s41598-019-54522-2

**Published:** 2019-12-02

**Authors:** Danielle F. Vileigas, Victoria M. Harman, Paula P. Freire, Cecília L. C. Marciano, Paula G. Sant’Ana, Sérgio L. B. de Souza, Gustavo A. F. Mota, Vitor L. da Silva, Dijon H. S. Campos, Carlos R. Padovani, Katashi Okoshi, Robert J. Beynon, Lucilene D. Santos, Antonio C. Cicogna

**Affiliations:** 1 Department of Internal Medicine, Botucatu Medical School, São Paulo State University (UNESP), Botucatu, São Paulo 18618687 Brazil; 2 Centre for Proteome Research, Institute of Integrative Biology, University of Liverpool, Liverpool, Merseyside, L69 7ZB United Kingdom; 3 Department of Morphology, Institute of Biosciences, São Paulo State University (UNESP), Botucatu, São Paulo 18618970 Brazil; 4 Department of Biostatistics, Institute of Biosciences, São Paulo State University (UNESP), Botucatu, São Paulo 18618970 Brazil; 5 Center for the Study of Venoms and Venomous Animals (CEVAP)/Graduate Program in Tropical Diseases (FMB), São Paulo State University (UNESP), Botucatu, São Paulo 18610307 Brazil

**Keywords:** Biochemistry, Molecular biology, Cardiology, Endocrinology, Molecular medicine

## Abstract

Obesity is a pandemic associated with a high incidence of cardiovascular disease; however, the mechanisms are not fully elucidated. Proteomics may provide a more in-depth understanding of the pathophysiological mechanisms and contribute to the identification of potential therapeutic targets. Thus, our study evaluated myocardial protein expression in healthy and obese rats, employing two proteomic approaches. Male *Wistar* rats were established in two groups (n = 13/group): control diet and Western diet fed for 41 weeks. Obesity was determined by the adipose index, and cardiac function was evaluated *in vivo* by echocardiogram and *in vitro* by isolated papillary muscle analysis. Proteomics was based on two-dimensional gel electrophoresis (2-DE) along with mass spectrometry identification, and shotgun proteomics with label-free quantification. The Western diet was efficient in triggering obesity and impaired contractile function *in vitro*; however, no cardiac dysfunction was observed *in vivo*. The combination of two proteomic approaches was able to increase the cardiac proteomic map and to identify 82 differentially expressed proteins involved in different biological processes, mainly metabolism. Furthermore, the data also indicated a cardiac alteration in fatty acids transport, antioxidant defence, cytoskeleton, and proteasome complex, which have not previously been associated with obesity. Thus, we define a robust alteration in the myocardial proteome of diet-induced obese rats, even before functional impairment could be detected *in vivo* by echocardiogram.

## Introduction

Obesity is a complex metabolic disease that may lead to severe damage to health, and its prevalence worldwide has nearly tripled since 1975, according to the World Health Organization^1^. In 2016, over 650 million adults were obese, and if this current trend continues, it will reach increasingly alarming numbers^1,2^. Obesity has been considered a common global pandemic disease, imposing a heavy burden on public health with a profound impact on morbidity and mortality, and health care costs^3^.

Considerable clinical and experimental evidence has associated obesity with an increased risk for the development of various comorbidities^4^. Furthermore, obesity is recognized as an independent and increasingly prevalent risk factor for cardiovascular disease (CVD)^5^. The excess of adipose tissue may promote maladaptation that results in alterations in cardiac structure and systolic and, particularly, diastolic function, which subsequently may progress to heart failure depending on the severity and duration of obesity^6,7^. The mechanisms underlying functional impairment of the heart in obesity are complex and incompletely elucidated, and disturbances in pathways at the cardiomyocyte level, myocardium, and heart chambers may be involved^8^. Indeed, alterations in cardiac energy metabolism of the cardiomyocyte, particularly involving fatty acids, play a significant impact on cardiac function and efficiency, contributing to the development of obesity-related cardiomyopathies^9^.

The high level of technological advance in equipment and methods of analysis is leading to a more precise understanding of the mechanisms responsible for pathophysiological processes. One of the research approaches that has been highlighted is proteomics^10^. In cardiovascular medicine, proteomics aims to evaluate the changes in protein expression under disease conditions, to better understand their role in the pathophysiological basis of cardiac dysfunction. Such studies may stimulate the discovery of new diagnostic or prognostic biomarkers and the development of new therapeutic applications for combating heart disease^11^.

Many studies have investigated the heart proteome in different experimental and clinical non-obese models with atherosclerosis, myocardial ischemia, dilated cardiomyopathy, myocardial infarction, and heart failure^12–16^. However, alterations in the cardiac proteome related to obesity have been little explored. Some investigations were found in genetic models of obesity^17,18^ or dietary models using a high-fat diet^19–21^; such studies deployed different proteomic methods and were not always associated with cardiac dysfunction. Even in the absence of cardiac dysfunction in Western diet-induced obese animals, the cardiac proteome was subtly altered when assessed by label-free shotgun proteomics^22^. Other studies have evaluated changes in proteins associated with diastolic dysfunction of the heart that exhibit oxidative post-translational modification, via tandem mass-tagging approaches^23,24^.

Here, we aim to evaluate patterns of protein expression in the myocardium of obese rats induced by Western diet. For this purpose, we performed two different proteomic approaches: two-dimensional electrophoresis (2-DE) with mass spectrometry (MS) based-identification of differentially expressed spots, and shotgun proteomics followed by label-free quantification. The results of this study will aid understanding of the mechanisms inherent in cardiac remodelling in obesity.

## Materials and Methods

### Animal models and experimental protocol

Sixty‐day‐old male *Wistar* rats were housed in individual cages in a controlled environment under a 12-h light-dark cycle at a room temperature of 24 °C ± 2 °C and humidity of 55 ± 5%, with a free supply of food and water. Rats were randomly assigned to two groups (n = 13 for each group): control diet (C: 3.6 kcal/g among which 11% kcal from fat, 67% from carbohydrate, and 22% from protein) and Western diet (WD: 4.9 kcal/g among which 50% kcal from fat, 35% from carbohydrate, and 15% from protein). Each group was fed the respective diet for 41 weeks. The following ingredients were used in diets: corn bran, soybean hulls, soybean bran, dextrin, sucrose, fructose, soybean oil, palm kernel oil, palm oil, lard, salt, and vitamin and mineral complex. The control diet was custom-formulated with the same ingredients as the WD except for lard, palm oil, sucrose, and fructose, which were added only in the WD, and soybean oil, only in the control diet (Supplementary Table S1).

At the end of the experiment, the rats were fasted for 12 h, anesthetized (50 mg/kg ketamine; 10 mg/kg xylazine; i.p.), and euthanized by decapitation. Heart tissue was dissected, and then the left ventricle (LV) was removed and immediately freeze-clamped at the temperature of liquid nitrogen. Blood samples were collected, and the serum was separated by centrifugation at 1620 *g* for 10 min at 4 °C.

All the experiments were conducted following the Guide for the Care and Use of Laboratory Animals published by the National Research Council (2011) and approved by the Ethics Committee on Animal Experiments of the Botucatu Medical School, São Paulo State University (1169/2016-CEUA).

### Nutritional profile and assessment of the comorbidities associated with the obesity

The nutritional profile was evaluated according to the following parameters: food and caloric intake, feed efficiency, body weight, body fat, and adiposity index, as previously described^25^. Food intake and body weight were measured weekly. Caloric consumption was determined by multiplying the energy value of each diet (g × kcal) to the food intake. The feed efficiency was calculated, dividing the total body weight gain (g) by total energy intake (kcal) in order to analyze the animal’s capacity to convert consumed food energy in body weight. Total body fat was determined as the sum of epididymal, retroperitoneal, and visceral fat pad weights. The adiposity index was calculated as follows: (total body fat/final body weight) × 100.

To assess obesity comorbidities, the following parameters were measured: systolic blood pressure (SBP), oral glucose tolerance test (OGTT), homeostatic model assessment of insulin resistance (HOMA-IR) and serum lipid profile. All the analyses were evaluated as previously described^26^.

### Cardiac morphologic study

Macroscopic cardiac remodelling was determined by the following parameters: heart, atria and left and right ventricle weights, as well as their ratio with tibia length. Additionally, frozen LV samples were used for histologic analysis as previously described^27^. Briefly, LV transverse sections were cut at 5 µm thickness in a cryostat cooled to −20 °C and stained with hematoxylin and eosin to determine transverse myocyte diameter, which was measured in at least 50–70 myocytes from each LV as the shortest distance between borders drawn across the nucleus. Collagen interstitial fraction was also determined using picrosirius red staining of LV sections, and on average, 20 microscopic fields were used to quantify interstitial collagen fractional area. Perivascular collagen was excluded from this analysis. All the measurements were performed using a Leica microscope (magnification 40×) attached to a video camera and connected to a computer equipped with image analysis software (Image-Pro Plus 3.0, Media Cybernetics, Silver Spring, MD, USA).

### Echocardiographic study

Echocardiograms were performed before euthanasia using a commercially available echocardiography (General Electric Medical Systems, Vivid S6, Tirat Carmel, Israel) equipped with a 5–11.5 MHz multifrequency transducer as previously described^25,27^. Briefly, two‐dimensionally guided M-mode images were obtained from parasternal short‐axis views of the LV just below the tip of the mitral valve leaflets at the level of the papillary muscles, and at the level of the aortic valve and left atrium. The following LV structural parameters were evaluated: LV diastolic diameter (LVDD), LV diastolic posterior wall thickness (DPWT), LV relative wall thickness (RWT), and diameters of the left atrium (LA) and aorta (AO). LV function was assessed by the following parameters: endocardial fractional shortening (EFS), ejection fraction (EF), posterior wall shortening velocity (PWSV), Tissue Doppler imaging (TDI) of mitral annulus systolic velocity (S′), early and late diastolic mitral inflow velocities (E and A waves), isovolumic relaxation time (IVRT), E wave deceleration time (EDT), TDI of early mitral annulus diastolic velocity (E′), and myocardial performance index (Tei index).

### Myocardial functional analysis

The intrinsic myocardial contractile performance was evaluated by an isolated papillary muscle (IPM) study from LV, as previously described^28^. The following parameters were measured from isometric contraction: developed tension (DT; g/mm^2^), resting tension (RT; g/mm^2^), peak of positive (+dT/dt; g/mm^2^/sec), and negative (−dT/dt; g/mm^2^/sec) tension derivative. The mechanical behaviour of IPM was evaluated under baseline condition at 2.5 mM Ca^2+^ and after the inotropic manoeuvres: post-rest contraction (PRC) and extracellular Ca^2+^ concentration increase. All manoeuvre values were expressed as the mean percent of baseline data and were calculated as follows: D = (M2-M1)/M1 × 100, where M1 was the value in the baseline condition, and M2 was the value after the inotropic manoeuvres. All force data were normalized for muscle cross-sectional area (CSA). To avoid the central core hypoxia and impaired functional performance, IMP with CSA > 1.5 mm^2^ were excluded from the analysis.

### Proteomic analysis based on 2-DE followed by LC-MS/MS

For full experimental details of this analysis, see supplementary materials and methods. Briefly, the 2-DE analysis was performed using a pooled homogenized LV sample (11 animals in each group); the pool was analysed as three technical replicates. For the first dimension, the isoelectric electrophoresis focusing was performed with Immobiline DryStrip pH gradient 3–10 (13 cm in length) strips in an Ettan IPGphor 3 Isoelectric Focusing System (GE Healthcare). For the second dimension, the electrophoresis was carried out in a SE 600 Ruby electrophoresis systems (Ge Healthcare). Gels were scanned using Image Scanner III calibrated densitometer and analysed using Image Master 2D Platinum software (version 7.05, GE Healthcare). Protein spots with *p-*value < 0.05 and at least a 1.2-fold difference in abundance were considered as differentially expressed. These protein spots were manually cut from the gels, digested in-gel with trypsin (PROMEGA), and further identified by MS.

The MS analyses were performed using a quadrupole model mass spectrometer (MicrQ-TOF III; Bruker Daltonics) with an electrospray ionization source and coupled to a liquid chromatography (LC-20AT; Shimadzu). The MS data were processed by Bruker Data Analysis software (version 3.3, Bruker Daltonics) and analysed using Mascot v.2.1 (www.matrixscience.com) to identify the proteins. The following search settings were used: trypsin enzyme, one permitted miscleavage, 0.1 Da peptide tolerance, 0.1 Da fragment ion mass tolerance, methionine oxidation as a variable modification, carbamidomethylation of cysteine as a fixed modification, and *Rattus norvegicus* taxonomy in NCBI database (151,390 sequences; 75,214,998 residues – March 2017).

### Shotgun proteomics followed by label-free quantification

For full experimental details of this analysis, see supplementary materials and methods. Briefly, LV samples from eight individuals in each of the control and WD groups (non-pooled samples) were used to perform the analysis. For the in-solution digestion, a fixed amount of protein from each sample (100 μg) was initially incubated at 80 °C with RapiGest (0.05% w/v final concentration, Waters, Manchester, UK) and then digested using sequencing grade trypsin (Promega). The cleared peptide digests were analyzed using a Q-Exactive HF Hybrid quadrupole-Orbitrap mass spectrometer (Thermo Fisher Scientific, Hemel Hempstead, UK) coupled to a Dionex Ultimate 3000 RSLC nano-liquid chromatography (Thermo Fisher Scientific, Hemel Hempstead, UK). The MS raw data files were loaded into Progenesis QI for Proteomics v.4.0 (Nonlinear Dynamics, Waters, Newcastle upon Tyne, UK) to perform the quantitative analysis. The peak list was searched against the UniProt database of *Rattus norvegicus* using Mascot v.2.6 (Matrix Science, London, UK) (7,989 sequences; 4,044,314 residues – January 2018). Trypsin was the specified enzyme, and one missed cleavage was allowed. Carbamidomethylation of cysteine was set as a fixed modification and oxidation of methionine as a variable modification. A precursor mass tolerance of 10 ppm and a fragment ion mass tolerance of 0.01 Da were applied. The false discovery rate was set at 1%. The criteria to consider a protein significantly up- or down-regulated were: identification and quantification using at least two unique peptides and *q*-value < 0.05.

Protein expression of all proteomic data was displayed in a volcano plot according to their statistical *p*-value and their relative difference of abundance (i.e., fold change), using an online tool (https://paolo.shinyapps.io/ShinyVolcanoPlot/). The relative expression levels of the differentially expressed proteins across the experimental groups were visualized using heatmap generated with a web tool for visualizing the clustering of multivariate data ClustVis^29^. Unsupervised multivariate principal component analysis (PCA) was also built using ClustVis.

### Proteomic bioinformatics

All the differentially expressed proteins obtained from 2-DE and shotgun proteomic were subjected to enrichment analysis for the Gene Ontology (GO) terms “molecular function”, “biological process” and “cellular component” using Protein ANalysis THrough Evolutionary Relationships (PANTHER, v.13.1) bioinformatics tool (http://www.pantherdb.org)^30^. The protein-protein interaction networks were constructed using the online STRING database (https://string-db.org) version 11.0. All STRING network analyses were performed with a medium confidence level (0.4).

### Western blot analysis

To verify some of the differentially expressed proteins via 2-DE and shotgun proteomics, we conducted Western blot analysis for two proteins involved in lipid metabolism, as platelet glycoprotein 4 (Cd36) and fatty acid-binding protein (Fabp3). The following primary antibodies were used: anti-Cardiac Fabp (1:1000; ab133585, Abcam, Cambridge, MA, USA), and anti-CD36 (1:1000; ab133625). For full experimental details of this analysis, see supplementary materials and methods.

### Oxidative stress biomarkers analysis

Malondialdehyde (MDA) and protein carbonylation levels were measured as oxidative stress biomarkers in cardiac tissue. For full experimental details of this analysis, see supplementary materials and methods.

### Statistical analysis

Prior to further statistical analysis, all datasets were tested for normality using the Shapiro-Wilk test. Data are expressed as mean ± SD or median (Min-Max) and were subjected to Student’s *t*‐test or Mann-Whitney U-test for independent samples. The IPM function after the inotropic intervention was evaluated by ANOVA on the model of repeated measures for independent groups and complemented by the Bonferroni *post hoc* test for multiple comparisons. All tests performed were two-sided, and adjustments for multiple comparisons were applied where indicated. The level of significance considered was 5%. The statistical analyses were performed using SigmaPlot 12.0, and graphics were generated using GraphPad Prism 8.

## Results

### Effects of WD on nutritional, metabolic, and cardiovascular profiles

Prolonged exposure to WD caused increased final body weight, total body fat, and adiposity index, leading to obesity in the animals fed on this diet (Table 1). Although the food and caloric intake were lower, the feed efficiency was higher in the WD group, which led them to gain more weight than the control group (Table 1). The body weight showed a slight increase of 10% in the rats fed WD, while the adiposity index presented a significant rise of 85%. Therefore, the continuous feeding of a WD was efficient in promoting obesity in 41 weeks. Long-term WD-induced obesity led to significant cardiovascular and metabolic disorders. The SBP, AUC, HOMA-IR, insulin hormone, and serum levels of glucose and triacylglycerol were higher in the WD group than the control (Table 1). Thus, WD-fed rats recapitulated many features of metabolic syndrome, including glucose intolerance, insulin resistance, dyslipidaemia, and arterial hypertension. All these results are consistent with previous investigations^31,32^ and make the WD-fed animals an appropriate model to study obesity-linked complications.

**Table 1:** The nutritional profile and parameters associated with obesity.

Variables	Control (n = 13)	WD (n = 13)	*p-*value
Initial body weight, g	175 ± 26	177 ± 25	0.819
Final body weight, g	557 ± 50	613 ± 81	0.043
Total body fat, g	32.9 ± 8.4	68.6 ± 27.0	<0.001
Adiposity index, %	5.9 ± 1.2	10.9 ± 2.9	<0.001
Food intake, g	25.9 ± 2.3	16.7 ± 2.0	<0.001
Caloric consumption, kcal	93.4 ± 8.2	81.7 ± 9.6	0.003
Feed efficiency, %	1.43 ± 0.13	1.86 ± 0.14	<0.001
SBP, mmHg	125 ± 4	151 ± 23	0.027
Glucose, mg/dL	97 ± 9	109 ± 13	0.019
AUC, mg.dL^−1^.min	25,249 ± 2,908	32,140 ± 6,391	0.002
Insulin, ng/mL	0.92 ± 0.51	1.55 ± 0.61	0.011
HOMA-IR	5.7 ± 3.0	10.5 ± 4.3	0.006
Triacylglycerol, mg/dL	82 ± 35	109 ± 30	0.007
Total cholesterol, mg/dL	75 ± 14	71 ± 15	0.474
HDL, mg/dL	23 ± 3	24 ± 4	0.557

Values are means ± SD. Student’s *t*-test for independent samples. SBP: systolic blood pressure. AUC: area under the curve for glucose. HOMA-IR: homeostasis model assessment of insulin resistance. HDL: high-density lipoprotein.

### Effects of obesity on cardiac structure and function

The morphological study indicated that obese rats did not trigger cardiac hypertrophy and interstitial collagen accumulation since there was no significant difference between the groups in parameters as ratios of heart, atria and left and right ventricle weights with the tibia length, as well as transverse myocyte diameter and interstitial collagen fraction (Supplementary Table S2).

Regarding the echocardiography (Table 2), the data revealed only a statistically significant reduction in EFS and EF in the WD group after 41 weeks. Also, the PWSV showed a trend toward being decreased in the WD group (*p* = 0.089). Despite the significant decrease in EFS and EF, this change varied by 7% and 2%, respectively, indicating a modest alteration in the systolic function that may not reflect pathophysiologic significance. Although both systolic and diastolic dysfunctions have been found in obesity, several studies have been controversial in the dysfunction pattern^33,34^. Some authors also observed only significant decreased EF and/or EFS by echocardiography in obese animals^32,35^, in agreement with our results. It is noteworthy that cardiac performance *in vivo* depends on contractile muscle properties. However, several factors can also change the cardiac function, as preload, afterload, and heart rate, which suffer the influence of different stimulus as the hormonal and autonomic nervous systems^36^.

**Table 2:** Echocardiographic structural and functional data.

Variables	Control (n = 11)	WD (n = 11)	*p-*value
Heart Rate, bpm	235 ± 23	236 ± 23	0.978
LVDD, mm	7.98 ± 0.49	7.85 ± 0.35	0.507
DPWT, mm	1.38 ± 0.06	1.42 ± 0.05	0.129
RWT	0.35 ± 0.03	0.36 ± 0.02	0.174
LA/AO	1.37 ± 0.10	1.43 ± 0.07	0.102
EFS, %	55.7 ± 3.9	52.1 ± 3.3	0.030
EF	0.91 ± 0.02	0,89 ± 0,02	0.029
PWSV, mm/s	43.9 ± 5.5	40.5 ± 3.1	0.089
S’, cm/s	3.91 ± 0.31	4.08 ± 0.29	0.191
E, cm/s	77.3 ± 6.1	76.5 ± 8.3	0.816
A, cm/s	52.4 ± 7.4	50.7 ± 5.2	0.554
E/A	1,50 ± 0,19	1,52 ± 0,17	0.785
IVRT, ms	27.5 ± 3.3	28.6 ± 2.8	0.413
EDT, ms	58.0 ± 8.4	53.8 ± 3.7	0.158
E’, cm/s	4.51 ± 0.66	4.70 ± 0.38	0.423
E/E’	17.4 ± 2.9	16.4 ± 2.5	0.379
Tei index	0.44 ± 0.05	0.47 ± 0.03	0.186

Values are means ± SD. Student’s *t*-test for independent samples. LVDD, left ventricle (LV) diastolic diameter; DPWT, LV diastolic posterior wall thickness; RWT, relative wall thickness; LA, left atrial diameter; AO, aortic diameter; EFS, endocardial fractional shortening; EF, ejection fraction; PWSV, posterior wall shortening velocity; S’, tissue Doppler Imaging (TDI) of systolic velocity of the mitral annulus; E and A, early and late diastolic mitral inflow velocities, respectively; IVRT, isovolumic relation time; EDT, E wave deceleration time; E’, TDI of early mitral annulus diastolic velocity; Tei index, myocardial performance index.

**Figure 1:**
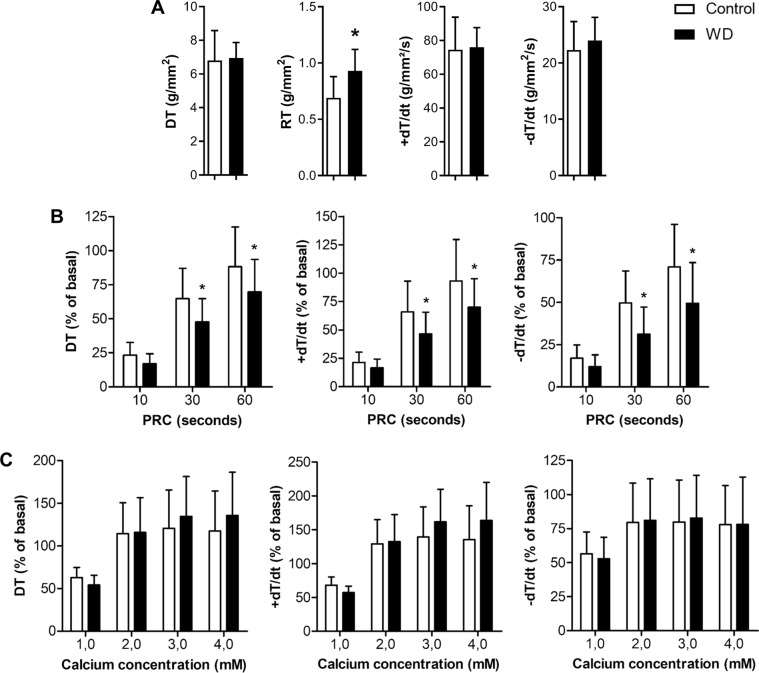
Left ventricular papillary muscle study. (**A**) Baseline condition, (**B**) post‐rest contraction and (**C**) effects of increasing extracellular Ca^2+^ concentration in isolated papillary muscle from control and Western diet (WD) groups (n = 11/group). DT, developed tension (g/mm^2^); RT, resting tension (g/mm^2^); +dT/dt, peak of positive tension derivatives (g/mm^2^/sec); −dT/dt, peak of negative tension derivatives (g/mm^2^/sec). All parameters normalized per cross-sectional area. Data are means ± SD. Student’s *t*-test for independent samples in (**A**) and repeated-measures two-way ANOVA and Bonferroni *post hoc* test in (**B**) and (**C**). *p < 0.05 vs. control.

Thus, we also evaluated the myocardial function *in vitro* by studying IPM at baseline and after inotropic manoeuvres (Fig. 1), which detects changes in the contractility of IPM, even with controlled afterload, preload, heart rate, energetic substrate and without hormonal and nervous system influence. The CSA of IPM did not differ between the groups (control = 1.01 ± 0.21, WD = 1.13 ± 0.22 mm^2^; *p* = 0.161). The IPM study, at baseline, evidenced diastolic dysfunction due to raised resting tension in the WD group (Fig. 1A), suggesting increased myocardial stiffness, which may be caused by elevation of collagen and cytosolic Ca^2+^ concentration, and/or ATP deficit^37^. In the current study, no rise in myocardial collagen was observed in the WD group, implying that the increased myocardial stiffness was probably due to impaired Ca^2+^ and/or ATP signalling. However, it is noteworthy that although collagen is the predominant component of the cardiac extracellular matrix, there are other proteins, e.g., fibronectin, secreted protein acidic and rich in cysteine (SPARC), and collagen cross-linking, which have also been associated with myocardial stiffness^38–40^. Figure 1B indicates that PRC induced a positive inotropic response in control and WD groups after cessation of the stimulus. However, this effect was significantly diminished in the WD group after 30 and 60 secs in the parameters DT, +dT/dt, and −dT/dt, indicating both systolic and diastolic dysfunction. As the PCR manoeuvre evaluates the release and recapture of Ca^2+^ by the sarcoplasmic reticulum (SR), this behaviour observed in the WD group can be attributed to impairment in Ca^2+^ reuptake by SR Ca^2+^-ATPase (ATP2A2), Ca^2+^ release by ryanodine receptor (RYR2) or affinity of Ca^2^ binding in troponin C. The Ca^2+^ stimulation manoeuvre showed no difference in the systolic functional parameters between the groups (Fig. 1C); this fact suggests that the most probable hypothesis is that the RYR2 function and Ca^2+^ binding in troponin C are not damaged in the WD group. Taken together, the results suggest cardiac dysfunction due to possible damage in the proper functioning of ATP2A2.

The divergence of results using different methodologies is probably attributable to the sensitivity and biological environment in which the evaluations were performed. Despite the functional changes in the IPM study, they were not of sufficient intensity to cause cardiac dysfunction *in vivo*, as assessed by echocardiography.

### Identification of differentially expressed proteins using 2-DE-based proteomics

**Figure 2:**
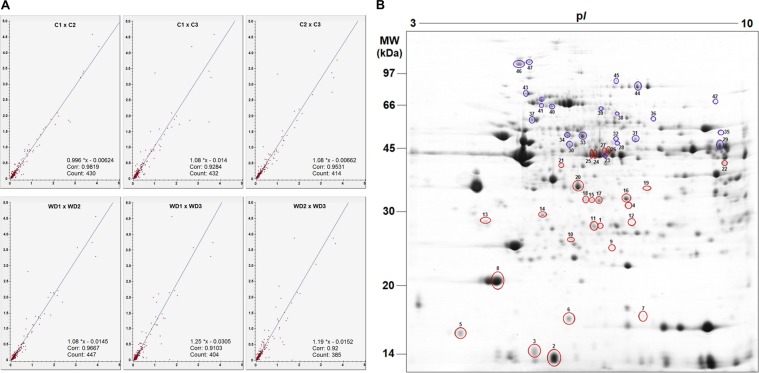
Proteomics data based on 2-DE of heart tissue from control (**C**) and Western diet (WD) groups. (**A**) Correlation analysis between 2-DE gels triplicates of control (**C**) and Western diet (WD) groups performed by Image Master 2D Platinum software. (**B**) Representative 2-DE gel image. The position of molecular weight (MW) markers are indicated to the right and the p*I* (isoelectric point) at the bottom of the gel. The sequence of numbers (1–47) refers to identification spots of the significantly up- (red circle) and down-regulated (blue circle) proteins in the WD group compared to the C group identified by LC-MS/MS. The detailed list of proteins for highlighted spots are shown in Supplementary Table S3.

The analysis showed that the triplicate 2D gels of the control and WD groups (Supplementary Fig. S1) were very similar, with correlation values greater than 0.90, ensuring reproducibility and quality, and reducing errors due to technical variations during electrophoresis (Fig. 2A). Software screening counted mean values of 490 and 503 spots respectively from control and WD 2‐DE gels. Most spots were resolved approximately in the 25–80 kDa molecular mass range and by the 5–9 pH area. The analysis revealed 47 significantly altered spots, and of these, we observed 27 increased, and 20 decreased in the WD group. A representative gel indicating the 47 spots is shown in Fig. 2B. Seven proteins, namely ECH1, ACADL, ACOT2, ENO1, ENO3, DLAT, MYH6, were identified in two resolved spots, possible protein isoforms or post-translational modifications, thus adjusting the total to 40 unique altered proteins. The detailed list of the 40 differently expressed proteins is provided in Supplementary Table S3.

### Identification of differentially expressed proteins using shotgun proteomic

The analysis allowed the identification and quantification of a total of 1226 proteins in the myocardial tissue extracts. This was reduced to 869 proteins by the addition of a further criterion of identification by a minimum of two unique peptides. Of these 869 proteins, 47 were significantly different between the groups (*q* < 0.05), with 23 and 25 proteins showing an increase or decrease in abundance, respectively, in the WD group relative to the control group (Supplementary Table S4).

**Figure 3:**
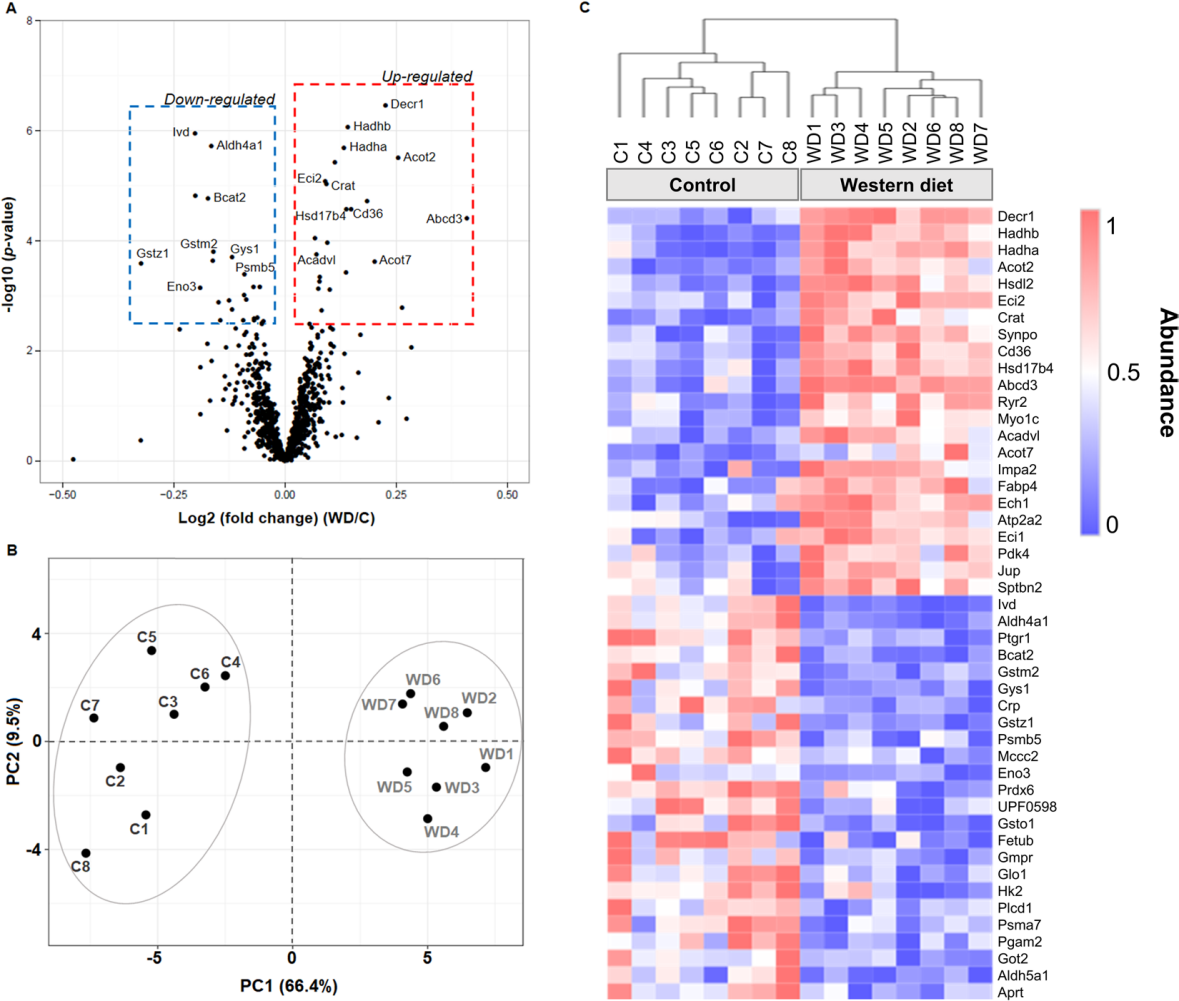
Label-free proteomics data clustering of heart tissue from control (**C**) and Western diet (WD) groups. (**A**) Univariate, significance (*p*-value) vs. fold change analyses highlighting several significant deregulated proteins of interest. (**B**) Unsupervised multivariate principal component analysis (PCA). (**C**) Hierarchical clustering analyses (Heatmap) using unsupervised Euclidean distance of all differentially expressed proteins between the groups. The detailed description of protein names is shown in Supplementary Table S4.

All data were visualised by a volcano plot to demonstrate the level of significance and magnitude of changes observed in the proteomic data, comparing the WD with the control group (Fig. 3A). The abundance of a small subset of proteins was markedly changed between the groups. Unsupervised PCA (Fig. 3B) and hierarchical clustering analysis (Fig. 3C) of all differentially expressed proteins show an evident separation between the two experimental groups, while there was some variance among the biological replicates.

### Biological functions of the differentially expressed proteins

We used two different proteomic approaches with distinct strengths and weaknesses to increase the breadth and confidence in the analysis. Combined, these analyses revealed 87 differentially expressed proteins in obese rat myocardium, and the list of the main proteins with their respective biological functions is summarized in Table 3. The two approaches identified different proteins, but most of them belong to the same functional category, and detected just five proteins in common (ACOT2, ECI2, ECH1, MCCC2, and ENO3) (Table 3). Indeed, our results demonstrated that the two methods (one based at the protein level, the other at the peptide level) are complementary, and using them in parallel may provide a more detailed understanding of the protein expression and molecular mechanisms of change.

**Table 3:** The biological function of the main up- and down-regulated proteins in the WD group compared to their controls identified by proteomics.

BIOLOGICAL FUNCTION	PROTEINS	
	Up	Down
**Lipid metabolism**		
Receptor and transport of fatty acids	CD36; FABP3; FABP4	—
Mitochondrial β-oxidation	DECR1; HADHB; HADHA; ECI1; ECI2* ACADVL; **ACADL**; ECH1*; **ECHS1**	—
Peroxisome β-oxidation	ABCD3; HSD17B4	—
Lipid Homeostasis	CRAT; ACOT2*; ACOT7	**ACSF2**
**Glucose metabolism**		
Glycolysis	ENO1	ENO3*; PGAM2; HK2
Glycogen catabolism	—	**PYGM**
Glycogen biosynthesis	—	GYS1
Conversion of pyruvate to acetyl-CoA	PDK4	**PDHA1**; **DLAT**
**Amino acid metabolism**		
BCAA oxidation	HIBADH	BCAT2; MCC2*; IVD
Aspartate biosynthetic process	—	GOT2
Proline catabolic process to glutamate	—	ALDH4A1
Malate-aspartate shuttle	—	SLC25A12
**Tricarboxylic acid cycle**	**MDH1**	**IDH2**; **ACO2**; ALDH5A1
**Oxidative phosphorylation**		
Electron transport chain	COX5B; ETFA	NDUFS1; NDUFS2; ETFDH
ATP-synthase complex	ATP5F1D	ATP5F1A
**Energy transduction**	—	**CKM**
**Antioxidant defence**	**PRDX3; PRDX5; SOD1; GSTA4**	PRDX6; GLO1; GSTM2; GSTO1; GSTZ1
**Proteasome complex**	—	PSMB5; PSMA7
**Cytoskeleton**	SPTBN2; JUP; SYNPO; **FHL2**; MYO1C	—
**Contraction**		
Contractile proteins	MYL2	MYH6
Regulatory proteins of Ca^2+^ handling	ATP2A2; RYR2	—

BCAA: branched-chain amino acids. The detailed description of protein names is shown in Supplementary Tables S3 and S4. *Equally detected by proteomic based on 2-DE gel and shotgun approach. Proteins typed in bold font were detected by 2-DE gel, and not bold font ones, by shotgun approach.

**Figure 4:**
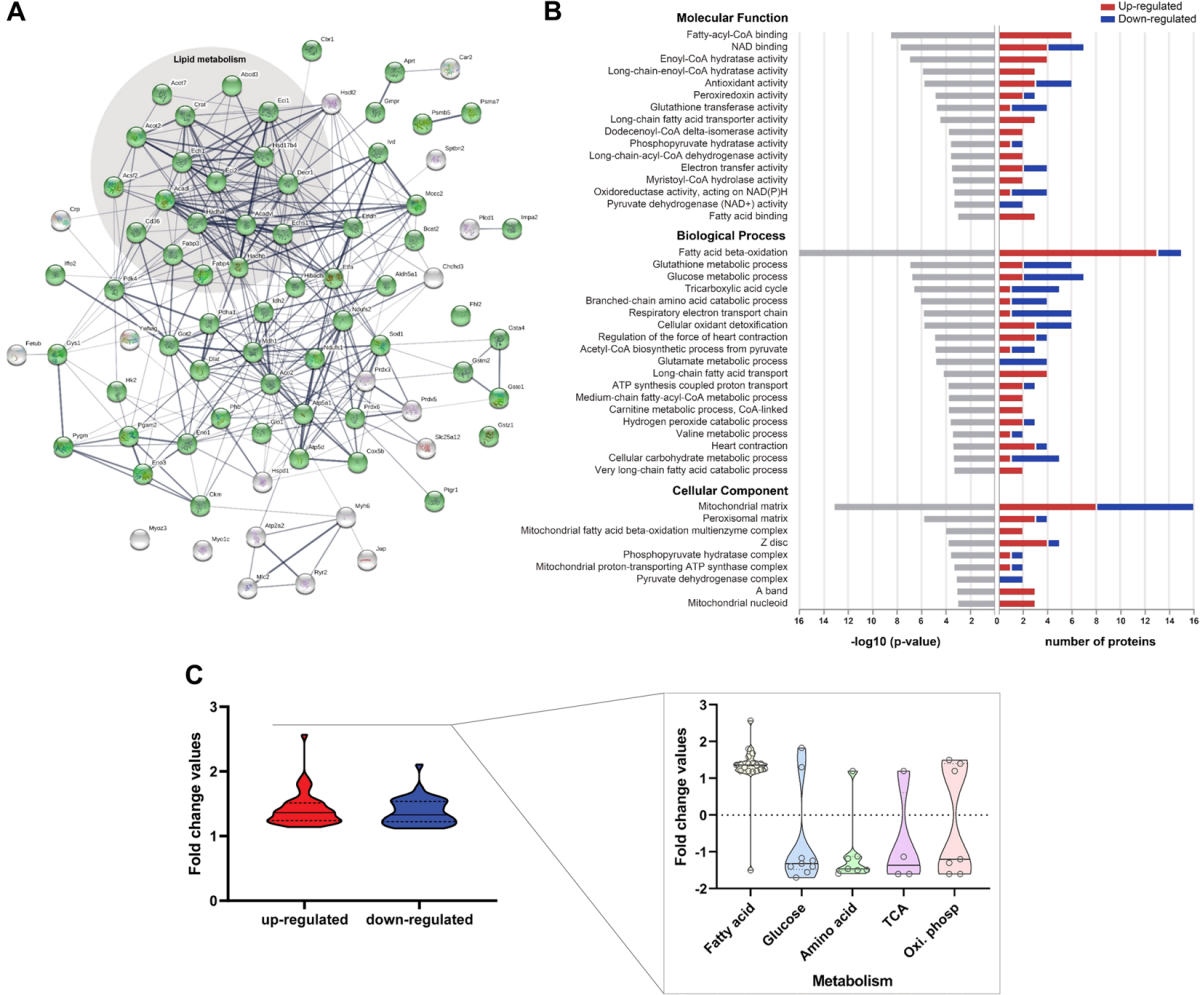
Biological functions of the differentially expressed proteins in the myocardium of obese rats identified by proteomics (**A**) Protein-protein interaction network. The interaction network analyses were built using the STRING online software with a medium confidence level (0.4). The circles represent proteins, while the straight lines represent the interactions between different proteins. The line thickness indicates the strength of evidence, with thicker connections indicating higher confidence in the protein-protein interaction. Green circles represent the proteins involved in metabolic processes; shaded area delimits the proteins involved in lipid metabolic processes. (**B**) Gene ontology (GO) enrichment analysis. The analysis was performed using the PANTHER tool (http://www.pantherdb.org), providing the significantly enriched GO terms Molecular Function, Biological Process, and Cellular Component. On the left panel, the horizontal axis indicates the significance (−log10 *p*-value) of the functional association, which is dependent on the number of proteins in the class. On the right panel, changes are displayed as the number of proteins with increased or decreased levels (horizontal axis). (**C**) Violin plot of the fold changes for up- and down-regulated proteins in the WD group compared to their controls. The fold changes of up- and down-regulated proteins were further separated into the metabolic processes associated with fatty acid, glucose, amino acid, tricarboxylic acid (TCA) cycle, and oxidative phosphorylation (Oxi. Phosp.). The circles inside the plots represent the changed proteins. The detailed description of protein names is shown in Supplementary Tables S3 and S4.

Protein-protein interaction analysis linked most of the altered proteins, showing a relationship between them (Fig. 4A). The majority of the proteins with changed abundance in the myocardium of obese rats were related to metabolic processes, which are represented by green circles (false discovery rate: 9.71e-32). Moreover, the network analysis also highlighted strong bias towards lipid metabolism, being the functional category with the highest number of changed proteins.

The fold change of all altered proteins varied up to an approximately 3-fold difference in abundance when comparing WD with the control group (Fig. 4C). In respect of cardiac metabolism, obese animals displayed protein alterations involved in the oxidation of different energetic substrates used for the production of ATP. The increase of proteins implicated in fatty acid oxidation was accompanied by a reduction in the proteins associated with oxidation of glucose and amino acids. In addition, the WD group also showed a decrease in most proteins involved in the tricarboxylic acid (TCA) cycle, electron transport chain (ETC), and ATP-synthase complex (Fig. 4C).

GO enrichment analysis showed that the altered proteins were focused on distinct molecular functions. The activities involved in cellular metabolism, particularly in lipid metabolism, constituted the most prominent category in molecular function (Fig. 4B). Most of the enriched pathways for GO Biological Process emphasised lipid metabolism, highlighting fatty acid β-oxidation. The majority of proteins included in these biological processes displayed higher expression in obese animals. Those proteins that were reduced the most play a role in different biological processes, such as glutathione metabolism, ETC, glucose and glutamate metabolism, TCA cycle, cellular carbohydrate metabolism, and leucine catabolism. A similar number of proteins up- and down-expressed were involved in ATP synthesis process and antioxidant defences. Furthermore, most proteins in the cardiac contraction process were increased (Fig. 4B). Finally, GO Cellular Component analysis suggested that the majority of changed proteins are mitochondrial. Altered proteins also highlighted the peroxisome and sarcomere (Fig. 4B).

**Figure 5:**
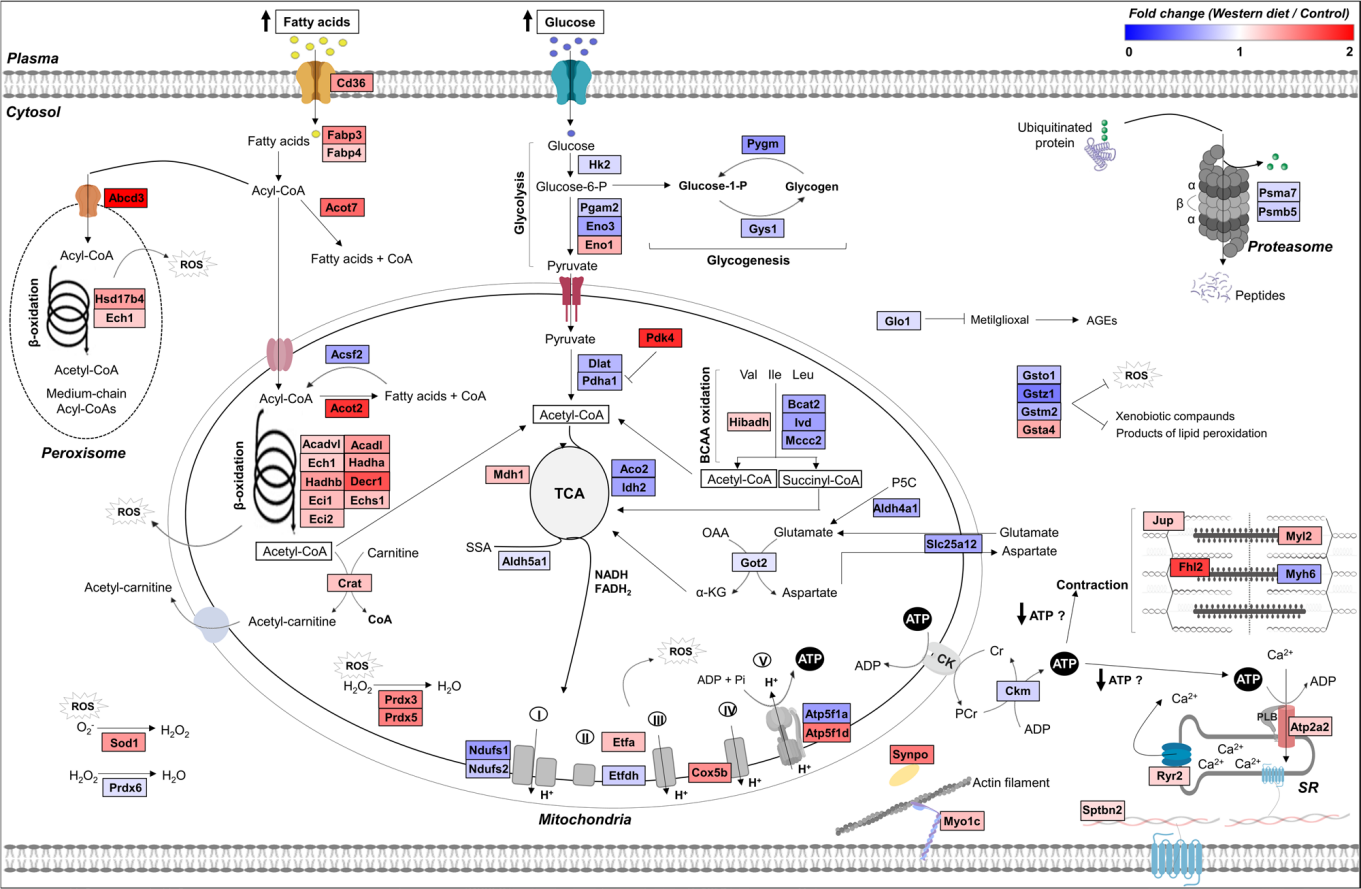
Overview of the obesity effects over cardiomyocyte proteome. The identified proteins are shown according to the magnitude of fold-change; red color for proteins up-regulated and blue color for proteins down-regulated in the myocardium of Western diet-induced obese rats compared to their controls. ROS, reactive oxygen species; AGEs, advanced glycation end products; Val, Ile, and Leu, valine, isoleucine, and leucine; BCAA, branched-chain amino acids; P5C, pyrroline-5-carboxylate; OAA, oxaloacetate; α-KG, α-ketoglutarate; TCA, tricarboxylic acid cycle; SSA, succinate semialdehyde; I, II, III, IV, and V, complexes of mitochondrial oxidative phosphorylation (I-IV: respiratory chain complexes; V: ATP synthase complex); CK, creatine kinase; Cr, creatine; PCr, phosphocreatine; SR, sarcoplasmic reticulum. The detailed description of protein names is shown in Supplementary Tables S3 and S4.

For better visualization of the key molecular alterations identified by the proteomic study inside the cardiomyocyte, Fig. 5 highlights these proteins according to the magnitude of their change (WD versus Control).

### Validation of proteomic data by protein expression profiles

**Figure 6:**
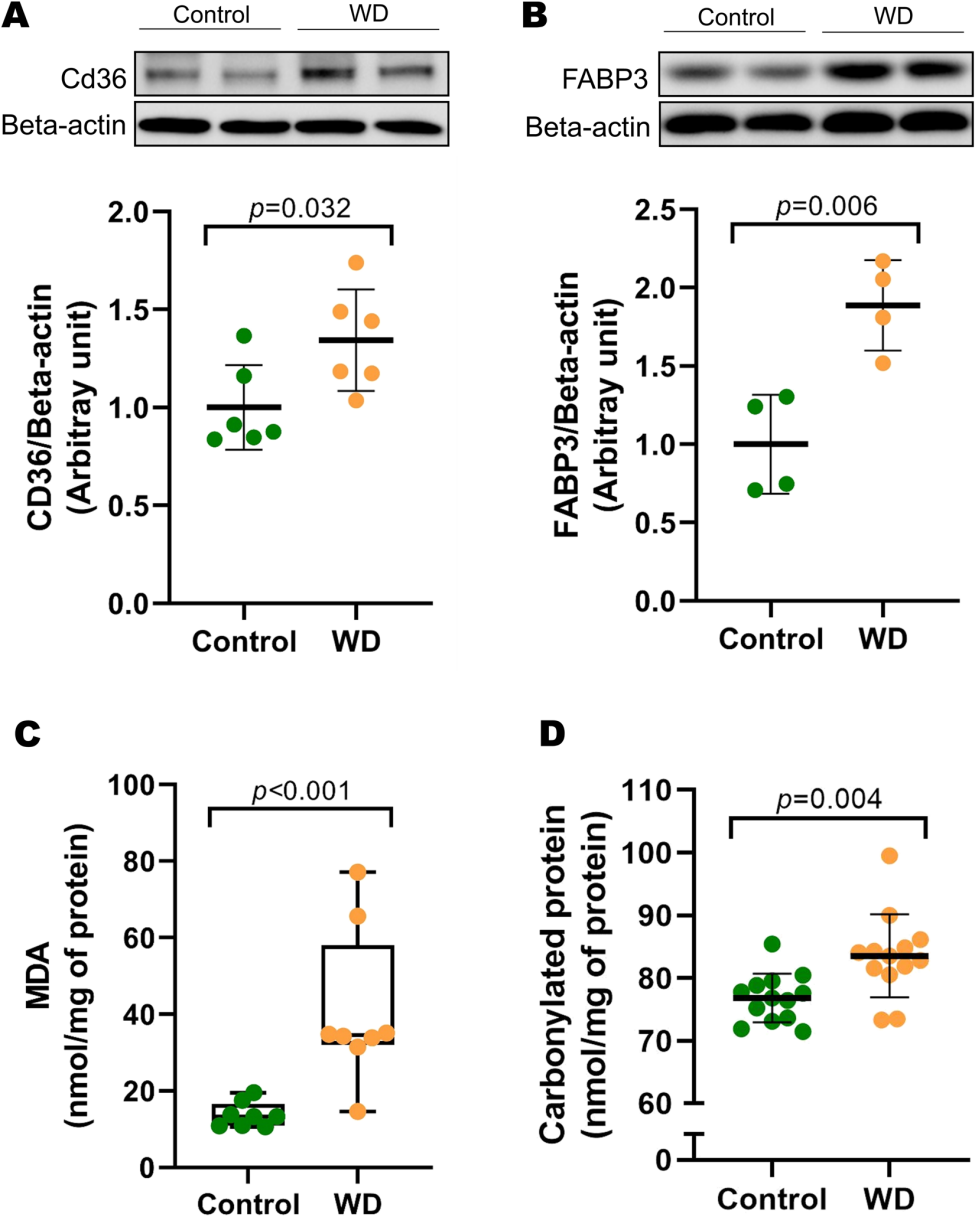
Pathways validation of proteomic data. The protein expression levels of (**A**) platelet glycoprotein 4 (CD36) and (**B**) fatty acid-binding protein (FABP3) were measured by Western blot and normalized to beta-actin (internal control) in myocardium from control and Western diet (WD) groups (n = 4–6/group). A representative Western blot and quantification of protein levels are shown. Images of blots have been cropped; the full-length blots are presented in Supplementary Fig. S2. Cardiac levels of (**C**) malondialdehyde (MDA) and (**D**) carbonylated protein of control and WD groups (n = 8–13/group). Values are mean ± SD or median (Min-Max). Student’s t‐test (in A, B, and D) or Mann-Whitney U-test (in C) for independent samples.

We performed western blot analysis to verify some of the differentially expressed proteins found by proteomics. We selected two critical proteins of lipid metabolism, such as Cd36 (identified by shotgun) and Fabp3 (identified by 2-DE), which play a role in the translocation of fatty acids across the sarcolemma of cardiac myocyte, and in fatty acids intracellular transport, respectively. The WD group showed higher protein expression for Cd36 and Fabp3 by Western blot (Fig. 6A,B), thus confirming the proteomic data.

### Oxidative stress biomarkers analysis

Metabolic alterations in obesity lead to increased reactive oxygen species (ROS) and oxidative stress. Based on the dysregulation of protein expression involved in antioxidant defence (Table 3 and Fig. 4B), which act to combat ROS, in the LV from obese rats, we hypothesized that indicators of oxidative stress such as MDA and protein carbonyl levels must be altered in these animals. The WD group presented higher values of MDA and protein carbonyl than the control group (Fig. 6C,D). Cardiac oxidative stress is thus evidenced by proteomics and by metabolite analyses.

## Discussion

A global molecular view of mechanisms related to cardiac remodelling due to obesity is not yet possible. Proteomics is a powerful technology that will provide new information about such biological mechanisms. Thus, we performed two different proteomic approaches to screen for protein expression changes in the left ventricle from control and obese rats. Overall, our results revealed a broad landscape of changed myocardial proteins and the involvement of several molecular networks in an obesity model, even before functional impairment could be detected *in vivo*. The proteomic analysis identified 87 differentially expressed proteins between the groups. The most changed proteins were involved in energy metabolism, oxidative stress, contractile and structural architecture, calcium transient, and proteolytic degradation via the proteasome complex.

### Proteins involved in energy metabolism

The most evident biological process was related to metabolism and, in particular, functions related to lipid metabolism. Despite the complex and multifactorial pathophysiology of heart damage related to excess adipose tissue, it has been suggested that modifications in cardiac energy metabolism are major contributors to cardiac dysfunction^41^. Obesity appears as a state of increased uptake and oxidation of fatty acids (FA) and reduced glucose utilization, which leads to abnormal use of cardiac energy, disturbance of myocardial efficiency, and compromised cardiac function^41,42^. This condition may occur initially due to high levels of circulating triglycerides (TG) and free FA and overexpression of FA transporters into cardiomyocytes. This is expected, as our study showed changes in FA uptake and both oxidation of FA and glucose in obese animals.

The capture of FA is highly active due to an increased supply of FA coming from TG in obese rats, which was accompanied by elevated protein expression of transporters such as CD36 and FABP3. The FABP4 isoform, expressed mainly in adipose tissues, was also elevated in the myocardium of obese rats. Interestingly, studies have identified FABP4 as a novel adipokine, and serum concentrations have been directly related to heart failure and CVD^43,44^. It is not clear if cardiomyocytes can internalize this protein and take part in the regulation of cellular responses^45^. However, a recent study demonstrated that FABP4 is expressed in the cardiomyocytes and can promote cardiac hypertrophy by activating ERK signal^46^. Our study shows a possible early expression of this protein even in the absence of hypertrophy signs, contributing in some way to the dysregulation of cardiac metabolic disorders.

One of the main metabolic fates of excess intracellular FA is mitochondrial oxidation, supported by the increased expression of enzymes involved in mitochondrial β-oxidation. FA β-oxidation rates are enhanced in hearts from mice subjected to diet-induced obesity^47^. In addition to elevated mitochondrial β-oxidation, our results suggest that this process is also active in peroxisomes, as the ABCD3 and HSD17B4 proteins are increased. Peroxisomes have also been proposed to exert a kind of “safeguard” of cellular survival against cell toxicity by an excess of cellular lipids, thus preventing an overload of mitochondrial β-oxidation and lipotoxicity^48,49^. Research on cardiac injury has focused almost exclusively on the mitochondria, even though comparable processes, such as ROS production and the degradation of lipids, are also carried out by peroxisomes and thus deserve further attention^50^.

Our findings also demonstrated that compartments of the cardiac cell in the WD group appear to be altered in an attempt to reduce metabolites originating from excess FA, in order to regulate lipid homeostasis within the cardiomyocyte. This response has occurred both within the mitochondria (CRAT, ACOT2, and ACSF2) and in the cytoplasm (ACOT7) (Fig. 5).

In addition to changes in FA and glucose metabolism, proteins related to amino acid metabolism were mostly decreased in the WD group. Amino acid catabolism plays a major role in the production of TCA cycle intermediates^51^ and this decrease may suggest compromised TCA cycle activity. A recent study showed decreased branched-chain amino acids (BCAA) oxidation in hearts from obese mice; however, the authors state that the mechanism for the reduction in oxidation rates is unclear because changes in BCAA oxidation proteins were not observed^52^. Our data presented alterations in four proteins of BCAA degradation, three of which displayed lower levels in obese animals, supporting the evidence of reduced BCAA oxidation by a decrease in catabolic enzymes. A rise in BCAAs and/or BCAA intermediates can stimulate cardiomyocyte growth (hypertrophy), alter autophagy, impair mitochondrial function, and trigger ROS formation, contributing to cardiac dysfunction^53^. Further studies are needed to determine the potential contribution of defective BCAA metabolism in obesity to cardiovascular impairment.

We observed a decreased abundance of proteins of the TCA cycle, ETC, and ATP-synthase complex in the myocardium of obese rats, consistent with impaired energy production. Despite higher β-oxidation, the low expression of these proteins points to a loss of mitochondrial function, which has been observed^54,55^. Conversely, we also observed increase in some proteins involved in the TCA cycle, ETC, and ATP-synthase complex. This imbalance in oxidative phosphorylation systems may perhaps be compromising the production of ATP in obese animals. These results are further supported in our study by the decreased level of creatine kinase (CKM), which provides ATP in the sarcoplasm.

### Proteins involved in oxidative stress

The release of ROS exceeding endogenous antioxidant capacity can indicate oxidative stress. Metabolic alterations observed in the obese heart, mainly increased FA oxidation, mitochondrial dysfunction, and glucose autoxidation raise ROS release; prolonged exposure or inappropriate subcellular localization of ROS may have detrimental cardiac effects^56^. Our proteomic data evidenced several changed proteins involved in antioxidant defence mechanisms against oxidative stress.

Two antioxidant enzymes located within the mitochondria (PRDX3 and PRDX5), responsible for ROS detoxifying mainly hydrogen peroxide (H_2_O_2_), presented higher levels in obese rats. In addition, we observed increased expression of two cytosolic enzymes (SOD1, GSTA4). These findings likely reflect a compensatory response to combat ROS elevation. Others have also reported increased antioxidant defences in obese hearts^57,58^.

Obese animals also showed low levels of enzymes involved in defence mechanisms. We observed reductions in the protein expressions of three different types of glutathione S-transferases (GSTM2, GSTO1, GSTZ1), whose role is to remove the endogenously produced free radicals and eliminate electrophilic xenobiotic compounds and products of lipid peroxidation such as 4-hydroxynonenal^59^. Previous work has shown decreased glutathione S-transferase enzyme in hearts from obese mice^60^. Another protein that presented lower levels was PRDX6, which plays an essential role in antioxidant defence and turnover of cellular phospholipids, providing a complete system for the repair of peroxidised cell membranes^61^. Additionally, in the WD group, Glyoxalase 1 (GLO1) was a down-regulated protein. This enzyme detoxifies methylglyoxal (MG), a potent precursor of advanced glycation end-products (AGEs). MG is mostly produced during glycolysis, however, hyperglycaemia, hypoxia, ischemia, inflammation, and oxidative stress, while simultaneously inhibiting the activity of GLO1, leading to intra- and extra-cellular accumulation of MG and hence AGEs. In emerging studies, GLO1 has been implicated in diabetic cardiomyopathy, coronary artery disease (CAD), and myocardial infarction^62–64^. A recent study has shown that a diet supplemented with bioactive compounds, capable of inducing increased expression of GLO1, produced improved metabolic and vascular health in overweight and obese subjects^65^.

Antioxidant defences act through the synergism between their components, some of them being more activated and others diminished depending on the location and intensity of the oxidative aggression. Taken together, our results showed a disruption in proteins related to antioxidant capacity, suggesting cardiac oxidative damage in obese rats. To confirm this hypothesis, we further evaluated oxidative stress biomarkers in LV, which were altered in the WD group. Therefore, the dysregulation of the protein expressions involved in antioxidant defence has not been sufficient to contain the pro-oxidant state found in obesity.

### Proteins involved in structural architecture and contractile function

The cardiac cytoskeleton has recently emerged as a crucial player for maintenance of myocyte integrity. In addition to providing mechanical support, these structures are critical for contractile function, tension sensing, and signal transduction, as well as support for networks for transport of molecules and organelles^66^. Disorders in cytoskeletal components have been strongly associated with cardiac pathologies^67^.

Our study revealed a higher protein abundance of the cytoskeleton in obese animals, as SPTBN2, JUP, SYNPO, FHL2, and MYO1C. In the heart, the SPTBN2 and JUP have established roles in mechanical function, and alterations in these proteins are associated with heart failure and cardiomyopathy^68–70^. However, the roles of SYNPO, FHL2, and MYO1C proteins are less well understood. The SYNPO protein was first discovered as an actin-associated protein in kidney podocytes and postsynaptic densities of telencephalic synapses^71^. Two proteins from the synaptopodin family, namely myopodin/synaptopodin 2 and synaptopodin 2-like protein/CHAP, are highly expressed in the sarcomere Z-disc of the heart muscle^72,73^. The SYNPO family remains poorly understood, and it was not possible to identify which family belongs to the SYNPO identified in our study. Regarding the FHL2, little prior work has evaluated its role in cardiovascular disease; however, evidence indicates that *FHL2* inhibits hypertrophic pathways of rat myocytes^74^, and it is associated with autophagy in mouse aortic endothelial cells^75^. Finally, the MYO1C, unconventional myosin, is an actin-associated molecular motor protein involved in the transport of vesicular cargo, including the trafficking of Glut4 glucose transporter, epithelial Na + channel, podocyte protein Neph1, and NF-κB essential modulator^76–78^. Recent studies have shown novel knowledge regarding the function of MYO1 in the dynamic regulation of cellular processes in different tissues and cells^79,80^, but its role in cardiac injury remains unknown.

Despite the fact that the changes in cytoskeleton components have already been found in obese hearts, such as titin and desmin^22,81^, interestingly, our data highlighted alterations in several cytoskeletal proteins not previously associated with obesity, providing novel and remarkable insights into the functioning of the cytoskeleton in this pathological condition.

We also observed altered proteins involved in the contractile function. The MYL2 showed higher expression in obese rats, while MYH6 was decreased. Reduced MYH6 and MYL2 have been found in hearts from obese animals with functional damage^82,83^, partially corroborating our data.

### Proteins involved in myocardial Ca^2+^ handling

A higher abundance of intracellular Ca^2+^-cycling proteins, the sarcoplasmic reticulum Ca^2+^-ATPase (ATP2A2) and ryanodine receptor (RYR2), was also observed in obese animals. These proteins play an essential role in regulating myocardial contraction/relaxation by regulating calcium transient homeostasis. Thus, changes in proteins involved in coordinating Ca^2+^ movement may contribute to contractile dysfunction^84^. The cardiac dysfunction in papillary muscle analysis evidenced possible damage to the proper functioning of ATP2A2. We hypothesize that the compromise in ATP production can lead to the inadequate functioning of this protein since it is ATP-dependent. Further, oxidative stress due to enhanced ROS production could induce oxidative post-translational modifications (PTMs) in ATP2A2, leading to depressed activity^85^. Functional damage in this protein may have triggered high expression as a compensatory mechanism; however, this elevation appears to be inadequate to maintain the heart function properly. Impairment in Ca^2+^ reuptake by ATP2A2 can decrease the concentration of this ion in SR and, consequently, the amount available for release, via RYR2, during systole^37^; this may be the possible explanation for the increased protein expression of RYR2 in the obese group in order to potentiate the Ca^2+^ release from RS.

### Proteins involved in proteasome complex

Our proteomics data showed increased proteasome proteins, as PSMB5 and PSMA7, in obese animals. Proteasomes are responsible for the degradation of intracellular protein, including unneeded or damaged proteins. The cardiac proteasome contains different proportions of subunits, and alterations in their composition affect overall proteasome proteolytic activity leading to the accumulation of damaged or misfolded protein. Such protein accumulation can contribute to pathogenesis in cardiomyopathies, as seen in non-obese models^86,87^. Impaired proteasomal degradation may be a feature of late stages of obesity and diabetes since failing hearts from chronically obese humans with type-2 diabetes accumulate of non-degraded protein^88,89^. Interestingly, our findings support this theory and provide new insights regarding the changed proteasome complex in obesity.

## Conclusions

In conclusion, the WD model was effective in promoting obesity and features of metabolic syndrome. Moreover, our findings showed that although myocardial functional study *in vitro* revealed impaired contractile function, analysis *in vivo* did not display cardiac dysfunction in obese rats. The combination of two proteomic approaches provided broader results, allowing the identification of an important number of altered myocardial proteins in a model of diet-induced obesity. These proteins are involved in critical biological processes, mainly in energy metabolism. These data will help develop novel hypotheses, but future studies are needed to elucidate the role of these changed proteins in cardiac remodelling in obesity.

### Note

For all protein symbols obtained from proteomic study and cited throughout the text, please, check their descriptions in the Supplementary Tables S3 and S4.

## Supplementary information

Supplementary Information

## Data Availability

The mass spectrometry proteomics data in this study are available in the following databases: Proteomic based on 2-DE: Atlas Peptides Repository from Institute for System Biology (http://www.peptideatlas.org) with the dataset identifier PASS01359. Proteomic based on shotgun: ProteomeXchange Consortium via the PRIDE partner repository (https://www.ebi.ac.uk/pride/archive) with the dataset accession number PXD013543 and 10.6019/PXD013543.
