# Supplementary material for: Landscape of heart proteome changes in a diet-induced obesity model
Source: Sci Rep. 2019 Dec 2;9:18050. doi: 10.1038/s41598-019-54522-2 (PMC6888820; doi:10.1038/s41598-019-54522-2)
Supplement: Supplementary file 1 — Supplementary Information [file 41598_2019_54522_MOESM1_ESM.docx]

**Landscape of heart proteome changes in a diet-induced obesity model**

Danielle F. Vileigas^1^*, Victoria M. Harman^2^, Paula P. Freire^3^, Cecília L. C. Marciano^1^, Paula G. Sant’Ana^1^, Sérgio L. B. Souza^1^, Gustavo A. F. Mota^1^, Vitor L. Silva^1^, Dijon H. S. Campos^1^, Carlos R. Padovani^4^, Katashi Okoshi^1^, Robert J. Beynon^2^, Lucilene D. Santos^5^, Antonio C. Cicogna^1^*

^1^ Department of Internal Medicine, Botucatu Medical School, São Paulo State University (UNESP), Botucatu, São Paulo, 18618687, Brazil.

^2^ Centre for Proteome Research, Institute of Integrative Biology, University of Liverpool, Liverpool, Merseyside, L69 7ZB, United Kingdom.

^3^ Department of Morphology, Institute of Biosciences, São Paulo State University (UNESP), Botucatu, São Paulo, 18618970, Brazil.

^4^ Department of Biostatistics, Institute of Biosciences, São Paulo State University (UNESP), Botucatu, São Paulo, 18618970, Brazil.

^5^ Center for the Study of Venoms and Venomous Animals (CEVAP)/Graduate Program in Tropical Diseases (FMB), São Paulo State University (UNESP), Botucatu, São Paulo, 18610307, Brazil.

*** Corresponding author:** Department of Internal Medicine, Medical School, São Paulo State University, 18618-687, Botucatu, São Paulo, Brazil.

E-mail addresses: [dani.vileigas@gmail.com](mailto:dani.vileigas@gmail.com) (D. F. Vileigas), [ac.cicogna@unesp.br](mailto:ac.cicogna@unesp.br) (A.C. Cicogna).

**Supplementary information**

**Supplementary Materials and Methods**

**Two-dimensional gel electrophoresis (2-DE) analysis followed by LC-MS/MS**

Left ventricle samples from control and WD groups (11 animals in each group) were homogenized in a lysis buffer (7 M urea, 2 M thiourea, 4% CHAPS, 1% DTT and 1% protease inhibitors [Sigma‐Aldrich, St. Louis, MO, USA]) using an Polytron Ultra Turrax® homogenizer (T25 Basic, Ika Works, Wilmington, NC, USA), followed by centrifugation at 40,000 x g for 60 minutes at 4ºC. The total protein quantification was determined using the Bradford method with Bio-Rad Protein Assay Dye Reagent Concentrate (Bio-Rad). The 2-DE analysis was performed using a pooled homogenized tissue samples (11 animals in each pool), and all samples contributed to the same protein amount to form their respective control and WD pool. Each pool was analysed as three technical replicates, resulting in six gels.

For the first dimension, the isoelectric electrophoresis focusing (IEF) was performed with Immobiline DryStrip pH gradient 3-10 (13 cm in length) strips (GE Healthcare). The strips were rehydrated overnight at room temperature in a solution containing sample (400 µg of protein), 0.5% IPG buffer pH 3-10 (GE Healthcare), and DeStreak rehydration solution (GE Healthcare). The rehydrated strips were placed into an Ettan IPGphor 3 Isoelectric Focusing System (GE Healthcare) for the first dimension separation. After focusing, the strips were incubated in two equilibration buffers for 15 min. The first equilibration buffer contained 6 M urea, 50 mM Tris-HCl pH 8.8, 30% glycerol, 2% SDS, 0.002% bromophenol blue and 1% DTT. The second equilibration buffer had the same composition, except 2.5% IAA was used instead of DTT.

For the second dimension, the equilibrated strips were placed on top of a 12.5% SDS–polyacrylamide gel electrophoresis and sealed with 0.5% agarose. Electrophoresis was carried out in a SE 600 Ruby electrophoresis systems (Ge Healthcare) at 15 mA/gel for 15 min and 40 mA/gel for 3 hours at 10ºC.

After electrophoresis, the protein spots were stained with Coomassie Brilliant Blue (0.025% Coomassie Brilliant Blue R-250, 50% ethanol, 5% acetic acid) for 1 hour. Gels were scanned using Image Scanner III calibrated densitometer (GE Healthcare) and analyzed using Image Master 2D Platinum software (version 7.05, GE Healthcare). Protein spots with *p-*value < 0.05 and at least a 1.2-fold difference in abundance were considered as differentially expressed. These protein spots of interest were selected for protein identification by mass spectrometry (MS).

Protein spots with differential intensity were manually cut from the gels and digested in-gel with trypsin, according to Shevchenko et al. (1) with some modification. Briefly, excised spots were first destained with acetonitrile (ACN)/25 mM ammonium bicarbonate (AMBIC) (1:1, v/v). After discarding the solution, gel spots were dehydrated twice with ACN for 10 min at room temperature. After removing and evaporating the residual ACN, the gel spots were submitted to reduction and alkylation with 10mM DTT and 55 mM IAA, respectively, and then dehydrated with ACN again. The dried gel pieces were incubated with 10ng/μL trypsin (Promega, Madison, WI, USA) in 50 mM AMBIC at 37°C overnight. Peptides were sequentially extracted from the gels initially in 5% formic acid (v/v) for 10 min, and then twice in 50% ACN (v/v) with 5% formic acid for 10 min. Extracts were dried in a vacuum centrifuge, and the peptides were dissolved in 13 µl 0.1% formic acid before MS identification.

The MS analyses were performed using a quadrupole model mass spectrometer (MicrQ-TOF III; Bruker Daltonics) with an electrospray ionization (ESI) source and coupled to liquid chromatography (LC-20AT; Shimadzu). The chromatographic separation was carried out through a C18 reverse-phase column (4.5 × 100 mm; 1.8 µm). The mobile phase consisted of solvent A (0.1% [v/v] formic acid) and solvent B (ACN with 0.1% [v/v] formic acid). Elution conditions were optimized in a gradient from 0 to 85% solvent B at a flow rate of 0.2 mL/min for 60 min. The total injection volume was 10 µL. The column and the automatic applicator of the sample were kept at 25°C and 10°C, respectively. The mass spectrometer was calibrated with the following parameters: 4.5 kV capillary voltage, 180°C drying temperature, and nitrogen flow of 6 L/min at 8 bar pressure. External calibration was performed using ESI-L Low Concentration Tuning Mix (Agilent Technologies). Mass spectra were acquired in m/z range 50 to 3000. The MS data were processed by Bruker Data Analysis software (version 3.3, Bruker Daltonics) and analyzed using Mascot v.2.1 ([www.matrixscience.com](http://www.matrixscience.com)) to identify the proteins. The following search settings were used: trypsin enzyme, one permitted miscleavage, 0.1 Da peptide tolerance, 0.1 Da fragment ion mass tolerance, methionine oxidation as a variable modification, carbamidomethylation of cysteines as a fixed modification, and *Rattus norvegicus* taxonomy in NCBI database (151,390 sequences; 75,214,998 residues – March 2017). The mass spectrometry proteomics data have been deposited to the Atlas Peptides Repository from Institute for System Biology (http://www.peptideatlas.org) with the dataset identifier PASS01359.

**Nano-liquid chromatography-tandem mass spectrometry (nLC-MS/MS) analysis and label-free quantification**

A label-free shotgun proteomic analysis was performed with left ventricle samples from eight individuals in each of the control and WD groups (non-pooled samples). Samples were homogenized in 50 mM AMBIC containing protease inhibitors (cOmplete, mini, EDTA-free, Roche Diagnostics, West Sussex, UK) using a bead beater homogenizer (Minilys®, Bertin Instruments, Montigny-le-Bretonneux, France). The total protein quantification was determined by the Bradford method using Coomassie® Plus Protein Assay Reagent (Thermo Scientific) and bovine serum albumin (BSA) as standards. For the in-solution digestion, a fixed amount of protein from each sample (100 μg) was initially incubated with RapiGest^TM^ (0.05% w/v final concentration, Waters, Manchester, UK) for 10 min at 80°C. The samples were then reduced with 3mM DTT for 10 min at 60°C, followed by alkylation with 9mM IAA for 30 min in the dark at room temperature. Reduced and alkylated proteins were then digested using sequencing grade trypsin (Promega) at an enzyme:protein ratio of 1:50 overnight at 37 °C with continuous mixing. After proteolysis, the peptide digests were subsequently acidified by the addition of TFA at a final concentration of 0.5% (v/v) followed by incubation for 45 min at 37°C to allow precipitation of the RapiGest™ SF Surfactant. The sample digests were then centrifuged at 17,200 x g for 45 min.

The cleared peptide digests were analyzed using a Q-Exactive HF Hybrid quadrupole-Orbitrap mass spectrometer (Thermo Fisher Scientific, Hemel Hempstead, UK) coupled to a Dionex Ultimate 3000 RSLC nano-liquid chromatograph (Thermo Fisher Scientific, Hemel Hempstead, UK). Digests (1 μL) from each sample were loaded onto a trap column (Acclaim PepMap 100 C18, 75 μm x 2 cm, 3 μm packing material, 100 Å) at 12 μL/min with an aqueous solution containing 0.1% (v/v) TFA and 2% (v/v) ACN. After 7 min, the trap column was set in-line with an analytical column (Easy-Spray PepMap® RSLC 50 cm length × 75 μm inner diameter, C18, 2μm, 100 Å) (Thermo Fisher Scientific). Peptides were loaded in 0.1% (v/v) formic acid and eluted with a linear gradient of 3.8 – 50% buffer B (HPLC grade acetonitrile 80%(v/v) with 0.1% (v/v) formic acid) over 90 min at 300nl/min, followed by a washing step (5 min at 99% solvent B) and an equilibration step (18 min at 3.8% solvent B). The column was maintained at 40°C, and the effluent introduced directly into the integrated nano-electrospray ionization source operating in positive ion mode. The mass spectrometer was operated in data-dependent mode with survey scans acquired at a mass resolution of 60,000 at m/z 200. Up to the top 16 most abundant isotope patterns with charge states +2, +3 and/or +4 from the survey scan were selected with an isolation window of 2.0 Th for fragmentation by higher energy collisional dissociation with normalized collision energies of 30%. The maximum ion injection times for the survey scan and the MS/MS scans were 100 and 45 ms, respectively, and the ion target value will be set to 1E6 for survey scans and 1E5 for the MS/MS scans. Repetitive sequencing of peptides was minimized through the dynamic exclusion of the sequenced peptides for 20 sec.

Mass spectrometry raw data files were loaded into Progenesis QI for Proteomics v.4.0 (Nonlinear Dynamics, Waters, Newcastle upon Tyne, UK) to perform the quantitative analysis. For alignment, a reference run was automatically selected, and the other runs were aligned to this reference run with an alignment score higher than 80%. The peak list was searched against the UniProt database of *Rattus norvegicus* using Mascot v.2.6 (Matrix Science, London, UK) (7,989 sequences; 4,044,314 residues – January 2019). Trypsin was the specified enzyme, and one missed cleavage was allowed. Carbamidomethylation of cysteine was set as a fixed modification and oxidation of methionine as a variable modification. A precursor mass tolerance of 10 ppm and a fragment ion mass tolerance of 0.01 Da were applied. The false discovery rate (FDR) was set at 1%. Relative quantification was based on unique peptides. The criteria to consider a protein significantly up- or down-regulated were: identification and quantification using at least two unique peptides and *q*-value < 0.05, calculated in Progenesis QI. The mass spectrometry proteomics data have been deposited to the ProteomeXchange Consortium via the PRIDE partner repository (https://www.ebi.ac.uk/pride/archive) with the dataset accession number PXD013543 and 10.6019/PXD013543.

Protein expression of all proteomic data was displayed in a volcano plot according to their statistical *p*-value and their relative difference of abundance (i.e., fold change), using an online tool (<https://paolo.shinyapps.io/ShinyVolcanoPlot/>). The relative expression levels of the differentially expressed proteins across the experimental groups were visualized using heatmap generated with a web tool for visualizing the clustering of multivariate data ClustVis (2). Unsupervised multivariate principal component analysis (PCA) was also built using ClustVis.

**References**

1- Shevchenko A, Tomas H, Havlis J, Olsen J V, Mann M. In-gel digestion for mass spectrometric characterization of proteins and proteomes. Nat Protoc. 2006 Jan;1(6):2856–60.

2- Metsalu T, Vilo J. ClustVis: a web tool for visualizing clustering of multivariate data using Principal Component Analysis and heatmap. Nucleic Acids Res. 2015 Jul 1;43(W1):W566–70.

**Western Blot analysis**

LV proteins were extracted by homogenization in RIPA buffer containing protease (Sigma‐Aldrich, St. Louis, MO, USA) and phosphatase (Roche Diagnostics, Indianapolis, IN, USA) inhibitors using a bead beater homogenizer (Bullet Blender^®^, Next Advance, Inc., NY, USA), followed by centrifugation (12,000 x g, 4°C, 20 min). Protein concentrations were determined using the Pierce BCA Protein Assay Kit. A total of 25μg protein lysate was resolved by SDS-PAGE and transferred to nitrocellulose membrane (Armsham Biosciences, Piscataway, NJ). The blotted membrane was blocked with 5% non-fat dry milk in TBS-T (20 mmol/L Tris‐HCl pH 7.4, 137 mmol/L NaCl and 0.1% Tween 20) for 1h at RT and incubated overnight at 4 - 8°C with the following primary antibodies: anti-Cardiac Fabp (1:1000; ab133585, Abcam, Cambridge, MA, USA), and anti-CD36 (1:1000; ab133625). Then, blots were incubated for 1.5h at RT with secondary antibody (Abcam) followed by chemiluminescence detection with SuperSignal^®^ West Pico kit (Thermo Scientific) using ImageQuant™ LAS 4000 (GE Healthcare). Quantification of band intensities was performed using ImageJ software (NIH). Results were normalized to the β-actin (Cell Signaling).

**Oxidative Stress biomarkers analysis**

Malondialdehyde (MDA) and protein carbonylation levels was measured as oxidative stress biomarkers.

Malondialdehyde (MDA) concentrations was measured to estimate lipid peroxidation levels in cardiac tissue homogenate (1:10 in PBS, pH 7.4; centrifugation at 3000 rpm, 4ºC for 10 min). This method involves reaction of thiobarbituric acid (TBA) with the degradation product of lipid peroxidation, MDA, under conditions of high temperature and acidity to generate a coloured adduct that is measured spectrophotometrically at 535 nm (1). Briefly, 500 μL of buffer (0.67% TBA, 15% trichloroacetic acid, and 0.25 N HCL) was added to 200 μL sample, and mixture was centrifuged at 10000 rpm for 10 min. The supernatant was collected and heated in a boiling water bath (100ºC) for 45 min. After cooling, the absorbance readings were acquired using a spectrophotometer. The MDA concentration was obtained through the molar extinction coefficient (1.56 x 105 M^-1^ cm^-1^) and sample absorbance.

The protein carbonylation was measured by adapted method of Mesquita et al. (2), which is widely used to estimate carbonyl content in biological samples. The cardiac tissue homogenate (1:10 in PBS, pH 7.4; centrifugation at 3000 rpm, 4ºC for 10 min) was diluted 1:10 in PBS buffer. In 100 μL of sample were added 100 μL of 2,4-dinitrophenylhydrazine (10 mM DNPH in 2M HCl), and then incubated for 10 min at room temperature. Subsequently, 50 μL of 6M sodium hydroxide (NaOH) were added in the sample and incubated again for 10 min at room temperature. Absorbance was recorded at 450 nm using a spectrophotometer. The carbonylated protein content was achieved by absorbance measures and the molar extinction coefficient (22,000 M-1 cm-1).

**References**

1- Yagi K. A simple fluorometric assay for lipoperoxide in blood plasma. Biochem Med. 1976 Apr;15(2):212–6

2- Mesquita CS, Oliveira R, Bento F, Geraldo D, Rodrigues J V, Marcos JC. Simplified 2,4-dinitrophenylhydrazine spectrophotometric assay for quantification of carbonyls in oxidized proteins. Anal Biochem. 2014;458:69–71.

**Supplementary Table**

**Table S1.** Ingredients and nutritional composition of the control diet and Western diet fat (WDF).

| **Ingredients (g/kg)** | **Control** | **WDF** |
| --- | --- | --- |
| Soybean bran | 335 | 344 |
| Soybean hull | 189 | 117 |
| Corn bran | 278 | 80 |
| Dextrin | 147 | 20 |
| Fructose | -- | 100 |
| Sucrose | -- | 50 |
| Soybean oil | 14 | -- |
| Palm oil | -- | 40 |
| Palm kernel oil | 9 | 80 |
| Lard | -- | 140 |
| Salt | 4 | 8 |
| Vitamin and mineral premix | 25 | 25 |
|  |  |  |
| **Nutritional composition** |  |  |
| Carbohydrate (% kcal) | 67 | 35 |
| Fat (% kcal) | 10 | 50 |
| *Saturated* | *3.1* | *26.5* |
| *Unsaturated* | *6.9* | *23.5* |
| Protein (% kcal) | 23 | 15 |
| Calories (kcal/g) | 3.6 | 4.9 |

**Table S2.** Cardiac morphological evaluation.

| **Variables** | **Control (n=13)** | **WD (n=13)** | ***p* value** |
| --- | --- | --- | --- |
| Tibia, cm | 4.50 ± 0.07 | 4.52 ± 0.11 | 0.597 |
| HW/T, g/cm | 0.269 ± 0.024 | 0.281 ± 0.025 | 0.237 |
| ATW/T, g/cm | 0.025 ± 0.003 | 0.025 ± 0.003 | 0.699 |
| LVW/T, g/cm | 0.193 ± 0.018 | 0.198 ± 0.017 | 0.459 |
| RVW/T, g/cm | 0.052 ± 0.007 | 0.058 ± 0.010 | 0.085 |
| TMD, µm | 16.6 ± 1.2 | 15.7 ± 1.9 | 0.180 |
| Interstitial collagen, % | 6.27 ± 0.81 | 5.73 ± 0.93 | 0.153 |

Values are means ± SD. Student’s *t*-test for independent samples. HW, ATW, LVW, and RVW, heart, atria, left, and right ventricles weights (g), respectively; T, tibia; TMD, transverse myocyte diameter.

**Table S3.** List of significantly changed proteins in the left ventricles of hearts from control and Western diet groups identified by 2-DE followed by ESI-TOF MS/MS.

| **Spots** | **Accession number** | **Protein name** | **Gene**  **Symbol** | **pI / Mr (kDa)** | ***p-value*** | ***fold change**** | ***Score*** | ***Matches*** | **Sequence Coverage (%)** |
| --- | --- | --- | --- | --- | --- | --- | --- | --- | --- |
| 1 | D3ZUX5 | *Coiled‐coil‐helix‐coiled‐coil‐helix domain containing 3 (MICOS complex subunit)* | Chchd3 | 6.9 / 27.1 | 0.011 | 1.5 | 56 | 7/1 | 18 |
| 2 | P07483 | *Fatty acid-binding protein, heart* | Fabp3 | 5.9 / 13.5 | 0.0004 | 1.4 | 588 | 56/37 | 80 |
| 3 | P12075 | Cytochrome c oxidase subunit 5b, mitochondrial | Cox5b | 5.5 / 13.9 | 0.002 | 1.4 | 462 | 68/41 | 42 |
| 4 | P47727 | *Carbonyl reductase [NADPH] 1* | Cbr1 | 7.5 / 30.0 | 0.039 | 1.4 | 247 | 35/7 | 41 |
| 5 | P35434 | *ATP synthase subunit delta, mitochondrial* | Atp5d | 4.0 / 15.5 | 0.007 | 1.5 | 283 | 19/13 | 13 |
| 6 | P07632 | *Superoxide dismutase [Cu-Zn]* | Sod1 | 6.2 / 16.5 | 0.005 | 1.4 | 655 | 49/20 | 49 |
| 7 | Q9R063 | *Peroxiredoxin‐5, mitochondrial* | Prdx5 | 7.8 / 16.7 | 0.003 | 1.5 | 280 | 38/15 | 43 |
| 8 | P08733 | *Myosin regulatory light chain 2, ventricular/cardiac muscle isoform* | Myl2 | 4.7 / 20.1 | 0.003 | 1.3 | 908 | 98/67 | 85 |
| 9 | P14942 | *Glutathione S‐transferase alpha‐4* | Gsta4 | 7.2 / 23.8 | 0.049 | 1.3 | 215 | 54/6 | 51 |
| 10 | Q9Z0V6 | *Thioredoxin-dependent peroxide reductase, mitochondrial (peroxiredoxin-3)* | Prdx3 | 6.3 / 25.2 | 0.025 | 1.5 | 284 | 32/24 | 30 |
| 11 | P14604 | *Enoyl-CoA hydratase, mitochondrial* | Echs1 | 6.8 / 26.9 | 0.049 | 1.2 | 963 | 94/68 | 44 |
| 12 | P27139 | *Carbonic anhydrase 2* | Ca2 | 7.6 / 27.5 | 0.047 | 1.5 | 201 | 27/4 | 22 |
| 13 | P61983 | *14-3-3 protein gamma* | Ywhag | 4.5 / 27.9 | 0.001 | 1.9 | 658 | 71/47 | 55 |
| 14 | P67779 | *Prohibitin* | Phb | 5.7 / 28.7 | 0.025 | 1.3 | 1429 | 90/80 | 72 |
| 15 | P29266 | *3-hydroxyisobutyrate dehydrogenase, mitochondrial* | Hibadh | 6.8 / 31.2 | 0.016 | 1.2 | 958 | 79/61 | 54 |
| 16 | P13803 | *Electron transfer flavoprotein subunit alpha, mitochondrial* | Etfa | 7.5 / 31.2 | 0.022 | 1.2 | 749 | 72/44 | 70 |
| 17 | Q62651 | *Delta(3,5)-Delta(2,4)-dienoyl-CoA isomerase* | Ech1 | 6.9 / 30,9 | 0.001 | 1.5 | 780 | 71/46 | 52 |
| 18 | Q62651 | *Delta(3,5)-Delta(2,4)-dienoyl-CoA isomerase* | Ech1 | 6.6 / 31.2 | 0.010 | 1.4 | 176 | 24/1 | 29 |
| 19 | O35115 | *Four and a half LIM domains protein 2* | Fhl2 | 7.9 / 33.3 | 0.014 | 1.8 | 54 | 19/0 | 24 |
| 20 | O88989 | *Malate dehydrogenase, cytoplasmic* | Mdh1 | 6.5 / 33.7 | 0.004 | 1.2 | 716 | 84/52 | 39 |
| 21 | Q5XIB3 | *[Protein ADP-ribosylarginine] hydrolase-like protein 1* | Adprhl1 | 6.1 / 39.0 | 0.032 | 1.3 | 649 | 66/39 | 30 |
| 22 | Q5XIC0 | *Enoyl-CoA delta isomerase 2, mitochondrial* | Eci2 | 9.6 / 38.7 | 0.046 | 1.3 | 557 | 52/34 | 35 |
| 23 | P00564 | *Creatine kinase M-type* | Ckm | 7.1 / 41.1 | 0.033 | -1.2 | 968 | 106/60 | 43 |
| 24 | P15650 | *Long-chain specific acyl-CoA dehydrogenase, mitochondrial* | Acadl | 6.8 / 41.2 | 0.045 | 1.4 | 1503 | 119/71 | 52 |
| 25 | P15650 | *Long-chain specific acyl-CoA dehydrogenase, mitochondrial* | Acadl | 6.8 / 41.2 | 0.008 | 1.4 | 1269 | 112/68 | 50 |
| 26 | O55171 | *Acyl-coenzyme A thioesterase 2, mitochondrial* | Acot2 | 7.1 / 42.2 | 0.001 | 1.7 | 1111 | 86/61 | 41 |
| ***Spots*** | **Accession number** | **Protein name** | **Gene**  **Symbol** | **pI / Mr (kDa)** | ***p-value*** | ***fold change**** | ***Score*** | ***Matches*** | **Sequence Coverage (%)** |
| 27 | O55171 | *Acyl-coenzyme A thioesterase 2, mitochondrial* | Acot2 | 7.1 / 42.1 | 0.001 | 3.2 | 1079 | 83/65 | 28 |
| 28 | P26284 | *Pyruvate dehydrogenase E1 component subunit alpha, somatic form, mitochondrial* | Pdha1 | 7.3 / 44.2 | 0.001 | -1.4 | 660 | 74/13 | 37 |
| 29 | P56574 | *Isocitrate dehydrogenase [NADP], mitochondrial* | Idh2 | 9.5 / 42.9 | 0.014 | -1.6 | 2134 | 172/108 | 55 |
| 30 | Q641Y2 | *NADH dehydrogenase [ubiquinone] iron-sulfur protein 2, mitochondrial* | Ndufs2 | 6.3 / 44.1 | 0.039 | -1.3 | 919 | 86/58 | 19 |
| 31 | P15429 | *Beta-enolase* | Eno3 | 7.7 / 45.7 | 0.014 | -1.6 | 1042 | 102/67 | 52 |
| 32 | P15429 | *Beta-enolase* | Eno3 | 7.3 / 46.0 | 0.003 | -1.6 | 164 | 47/3 | 34 |
| 33 | P04764 | *Alpha-enolase* | Eno1 | 6.6 / 47.4 | 0.010 | 1.3 | 684 | 91/53 | 66 |
| 34 | P04764 | *Alpha-enolase* | Eno1 | 6.3 / 47.9 | 0.017 | 1.2 | 636 | 76/46 | 61 |
| 35 | P15999 | *ATP synthase subunit alpha, mitochondrial* | Atp5a1 | 9.5 / 48.1 | 0.014 | -1.6 | 638 | 84/15 | 46 |
| 36 | Q5XIT9 | *Methylcrotonoyl‐CoA carboxylase beta chain, mitochondrial* | Mccc2 | 8.1 / 55.5 | 0.016 | -1.5 | 585 | 76/10 | 49 |
| 37 | P63039 | *60 kDa heat shock protein, mitochondrial* | hspd1 | 5.5 / 55.9 | 0.015 | -1.3 | 2123 | 176/125 | 55 |
| 38 | Q499N5 | *Acyl‐CoA synthetase family member 2, mitochondrial* | Acsf2 | 7.3 / 58.9 | 0.003 | -1.5 | 107 | 21/2 | 17 |
| 39 | Q6UPE1 | *Electron transfer flavoprotein‐ubiquinone oxidoreductase, mitochondrial* | Etfdh | 7.0 / 61.8 | 0.045 | -1.2 | 681 | 72/13 | 32 |
| 40 | P08461 | *Dihydrolipoyllysine-residue acetyltransferase component of pyruvate dehydrogenase complex, mitochondrial* | Dlat | 6.0 / 63.6 | 0.040 | -1.3 | 1091 | 114/77 | 38 |
| 41 | P08461 | *Dihydrolipoyllysine-residue acetyltransferase component of pyruvate dehydrogenase complex, mitochondrial* | Dlat | 5.7 / 64.6 | 0.022 | -1.4 | 525 | 70/14 | 29 |
| 42 | F1LX07 | *Calcium-binding mitochondrial carrier protein Aralar1* | Slc25a12 | 9.4 / 64.8 | 0.004 | -1.5 | 586 | 61/18 | 35 |
| 43 | Q66HF1 | *NADH-ubiquinone oxidoreductase 75 kDa subunit, mitochondrial* | Ndufs1 | 5.4 / 73.8 | 0.024 | -1.6 | 543 | 57/42 | 32 |
| 44 | Q9ER34 | *Aconitate hydratase, mitochondrial* | Aco2 | 7.8 / 77.9 | 0.041 | -1.6 | 1437 | 153/104 | 49 |
| 45 | P09812 | *Glycogen phosphorylase, muscle form* | Pygm | 7.3 / 84.0 | 0.036 | -1.7 | 735 | 140/70 | 47 |
| 46 | P02563 | *Myosin-6* | Myh6 | 5.3 / 104.2 | 0.044 | -1.5 | 2341 | 270/166 | 33 |
| 47 | P02563 | *Myosin-6* | Myh6 | 5.5 / 107.0 | 0.007 | -1.8 | 2155 | 268/152 | 34 |

# pI: isoelectric point; Mr: Molecular Weight. * Values of negative *fold-change* indicate lower protein expression in the obese group when compared to control, while positive values indicate higher expression in the obese group.

**Table S4.** List of significantly changed proteins in the left ventricles of hearts from control and Western diet groups identified by label-free nLC-MS/MS.

| **Accession number** |  | **Symbol** | **Protein name** | **Peptide count (unique peptides)** | **Confidence score** | **Sequence Coverage (%)** | ***p*-value** | ***q*-value** | ***fold change**** |
| --- | --- | --- | --- | --- | --- | --- | --- | --- | --- |
| Q64591 |  | Decr1 | 2,4-dienoyl-CoA reductase | 18 (16) | 1440 | 69 | <0.001 | <0.001 | 1.68 |
| Q60587 |  | Hadhb | Trifunctional enzyme subunit beta | 25 (25) | 1778 | 58 | <0.001 | <0.001 | 1.39 |
| Q64428 |  | Hadha | Trifunctional enzyme subunit alpha | 40 (39) | 3309 | 66 | <0.001 | <0.001 | 1.36 |
| O55171 |  | Acot2 | Acyl-coenzyme A thioesterase 2 | 17 (9) | 1530 | 43 | <0.001 | <0.001 | 1.80 |
| Q4V8F9 |  | Hsdl2 | Hydroxysteroid dehydrogenase-like protein 2 | 20 (20) | 1446 | 50 | <0.001 | <0.001 | 1.29 |
| Q5XIC0 |  | Eci2 | Enoyl-CoA delta isomerase 2 | 10 (10) | 956 | 45 | <0.001 | <0.001 | 1.23 |
| Q704S8 |  | Crat | Carnitine O-acetyltransferase | 15 (15) | 849 | 30 | <0.001 | <0.001 | 1.24 |
| Q9Z327 |  | Synpo | Synaptopodin | 2 (2) | 125 | 5 | <0.001 | 0.001 | 1.53 |
| P97852 |  | Hsd17b4 | Peroxisomal multifunctional enzyme type 2 | 10 (10) | 466 | 23 | <0.001 | 0.001 | 1.37 |
| Q07969 |  | Cd36 | Platelet glycoprotein 4 | 13 (13) | 843 | 35 | <0.001 | 0.001 | 1.41 |
| P16970 |  | Abcd3 | ATP-binding cassette sub-family D member 3 | 2 (2) | 46 | 6 | <0.001 | 0.002 | 2.57 |
| B0LPN4 |  | Ryr2 | Ryanodine receptor 2 | 93 (93) | 5516 | 28 | <0.001 | 0.004 | 1.17 |
| Q63355 |  | Myo1c | Unconventional myosin-Ic | 16 (16) | 950 | 22 | <0.001 | 0.005 | 1.25 |
| P45953 |  | Acadvl | Very long-chain specific acyl-CoA dehydrogenase | 27 (27) | 2544 | 50 | <0.001 | 0.007 | 1.18 |
| Q64559 |  | Acot7 | Cytosolic acyl coenzyme A thioester hydrolase | 2 (2) | 112 | 7 | <0.001 | 0.007 | 1.59 |
| Q8CIN7 |  | Impa2 | Inositol monophosphatase 2 | 4 (4) | 167 | 17 | <0.001 | 0.011 | 1.37 |
| P70623 |  | Fabp4 | Fatty acid-binding protein, adipocyte | 8 (7) | 661 | 49 | <0.001 | 0.012 | 1.20 |
| Q62651 |  | Ech1 | Delta(3,5)-Delta(2,4)-dienoyl-CoA isomerase | 15 (15) | 906 | 65 | <0.001 | 0.014 | 1.20 |
| P11507 |  | Atp2a2 | Sarcoplasmic/endoplasmic reticulum calcium ATPase 2 | 57 (56) | 5133 | 55 | <0.001 | 0.017 | 1.19 |
| P23965 |  | Eci1 | Enoyl-CoA delta isomerase 1 | 16 (16) | 1061 | 70 | <0.001 | 0.017 | 1.26 |
| O54937 |  | Pdk4 | Pyruvate dehydrogenase(acetyl-transferring) kinase isozyme 4 | 3 (3) | 136 | 10 | 0.002 | 0.031 | 1.83 |
| Q6P0K8 |  | Jup | Junction plakoglobin | 26 (24) | 1761 | 54 | 0.002 | 0.033 | 1.21 |
| Q9QWN8 |  | Sptbn2 | Spectrin beta chain, non-erythrocytic 2 | 6 (6) | 218 | 2 | 0.003 | 0.046 | 1.14 |
| P12007 |  | Ivd | Isovaleryl-CoA dehydrogenase | 17 (17) | 1217 | 62 | <0.001 | <0.001 | -1.59 |
| P0C2X9 |  | Aldh4a1 | Delta-1-pyrroline-5-carboxylate dehydrogenase | 20 (20) | 1329 | 53 | <0.001 | <0.001 | -1.46 |
| P97584 |  | Ptgr1 | Prostaglandin reductase 1 | 5 (5) | 455 | 21 | <0.001 | <0.001 | -1.59 |
| O35854 |  | Bcat2 | Branched-chain-amino-acid aminotransferase | 11 (11) | 721 | 34 | <0.001 | 0.001 | -1.49 |
| P08010 |  | Gstm2 | Glutathione S-transferase Mu 2 | 19 (9) | 1402 | 82 | <0.001 | 0.006 | -1.45 |
| A2RRU1 |  | Gys1 | Glycogen [starch] synthase, muscle | 14 (14) | 729 | 28 | <0.001 | 0.007 | -1.32 |
| **Accession number** |  | **Symbol** | **Protein name** | **Peptide count (unique peptides)** | **Confidence score** | **Sequence Coverage (%)** | ***p*-value** | ***q*-value** | ***fold change**** |
| P48199 |  | Crp | C-reactive protein | 2 (2) | 114 | 9 | <0.001 | 0.007 | -1.45 |
| P57113 |  | Gstz1 | Maleylacetoacetate isomerase | 7 (7) | 311 | 47 | <0.001 | 0.008 | -2.11 |
| P28075 |  | Psmb5 | Proteasome subunit beta type-5 | 3 (3) | 242 | 14 | <0.001 | 0.012 | -1.23 |
| Q5XIT9 |  | Mccc2 | Methylcrotonoyl-CoA carboxylase beta chain | 21 (21) | 1499 | 54 | <0.001 | 0.017 | -1.18 |
| O35244 |  | Prdx6 | Peroxiredoxin-6 | 17 (17) | 1211 | 81 | <0.001 | 0.017 | -1.14 |
| P15429 |  | Eno3 | Beta-enolase | 23 (13) | 2155 | 65 | <0.001 | 0.017 | -1.55 |
| Q642A4 |  |  | UPF0598 protein C8orf82 homolog | 4 (4) | 220 | 27 | <0.001 | 0.020 | -1.24 |
| Q9Z339 |  | Gsto1 | Glutathione S-transferase omega-1 | 5 (5) | 262 | 31 | 0,001 | 0.024 | -1.33 |
| Q9QX79 |  | Fetub | Fetuin-B | 6 (6) | 278 | 30 | 0,001 | 0.025 | -1.41 |
| Q9Z244 |  | Gmpr | GMP reductase 1 | 8 (7) | 475 | 33 | 0,002 | 0.032 | -1.31 |
| Q6P7Q4 |  | Glo1 | Lactoylglutathione lyase | 7 (7) | 328 | 47 | 0,002 | 0.042 | -1.15 |
| P27881 |  | Hk2 | Hexokinase-2 | 11 (8) | 639 | 30 | 0,003 | 0.043 | -1.17 |
| P10688 |  | Plcd1 | 1-phosphatidylinositol 4,5-bisphosphate phosphodiesterase delta-1 | 3 (3) | 94 | 6 | 0,003 | 0.043 | -1.40 |
| P48004 |  | Psma7 | Proteasome subunit alpha type-7 | 4 (4) | 215 | 21 | 0,003 | 0.043 | -1.17 |
| P16290 |  | Pgam2 | Phosphoglycerate mutase 2 | 14 (11) | 1135 | 70 | 0,003 | 0.043 | -1.24 |
| P00507 |  | Got2 | Aspartate aminotransferase | 26 (26) | 2193 | 60 | 0,003 | 0.044 | -1.12 |
| P51650 |  | Aldh5a1 | Succinate-semialdehyde dehydrogenase | 10 (10) | 674 | 32 | 0,003 | 0.046 | -1.13 |
| P36972 |  | Aprt | Adenine phosphoribosyltransferase | 9 (9) | 662 | 66 | 0,003 | 0.046 | -1.13 |

# * Values of negative *fold-change* indicate lower protein expression in the obese group when compared to control, while positive values indicate higher expression in the obese group.

**Supplementary Figure**

**Figure S1**


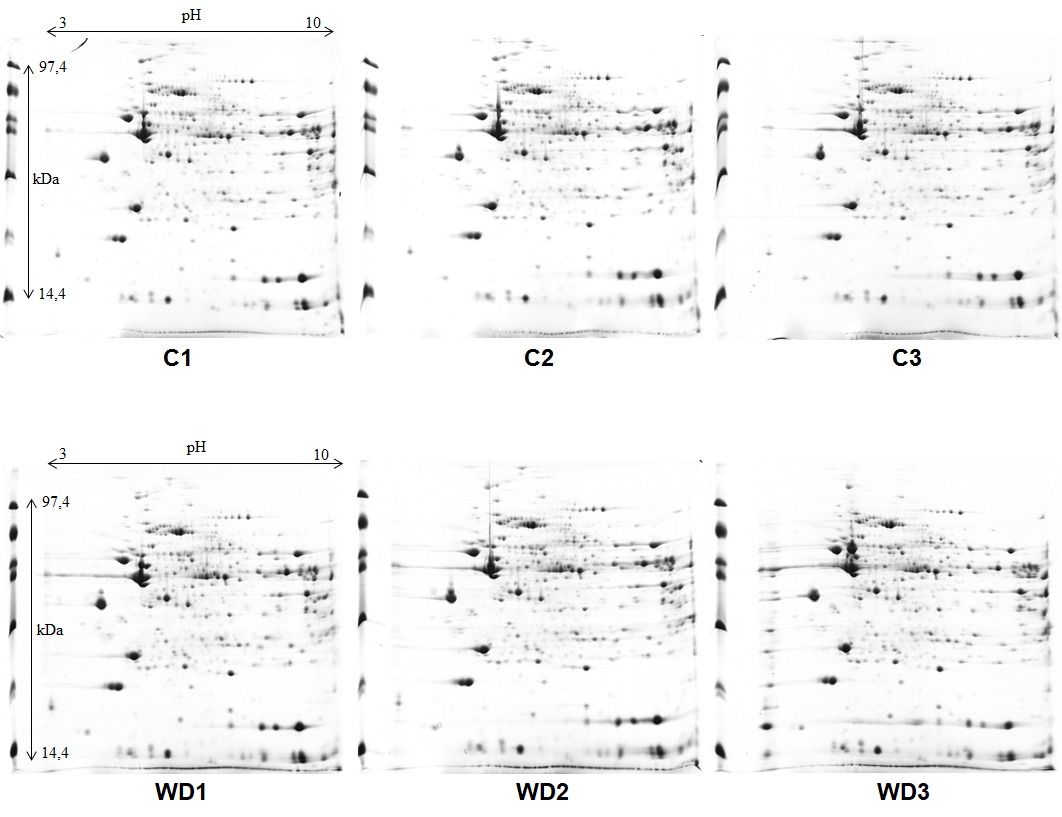


**Figure S1.** **Representative 2-DE gels of heart left ventricle from control (C) and Western diet (WD) groups in triplicate.** Gel spots were stained with Coomassie Brilliant Blue (0.025% Coomassie Brilliant Blue R-250, 50% ethanol, 5% acetic acid). Gels scanned using Image Scanner III calibrated densitometer (GE Healthcare).

**Figure S2**

Weight Marker

(Precision Plus Protein Standards Kaleidoscope™ - BioRad)

**
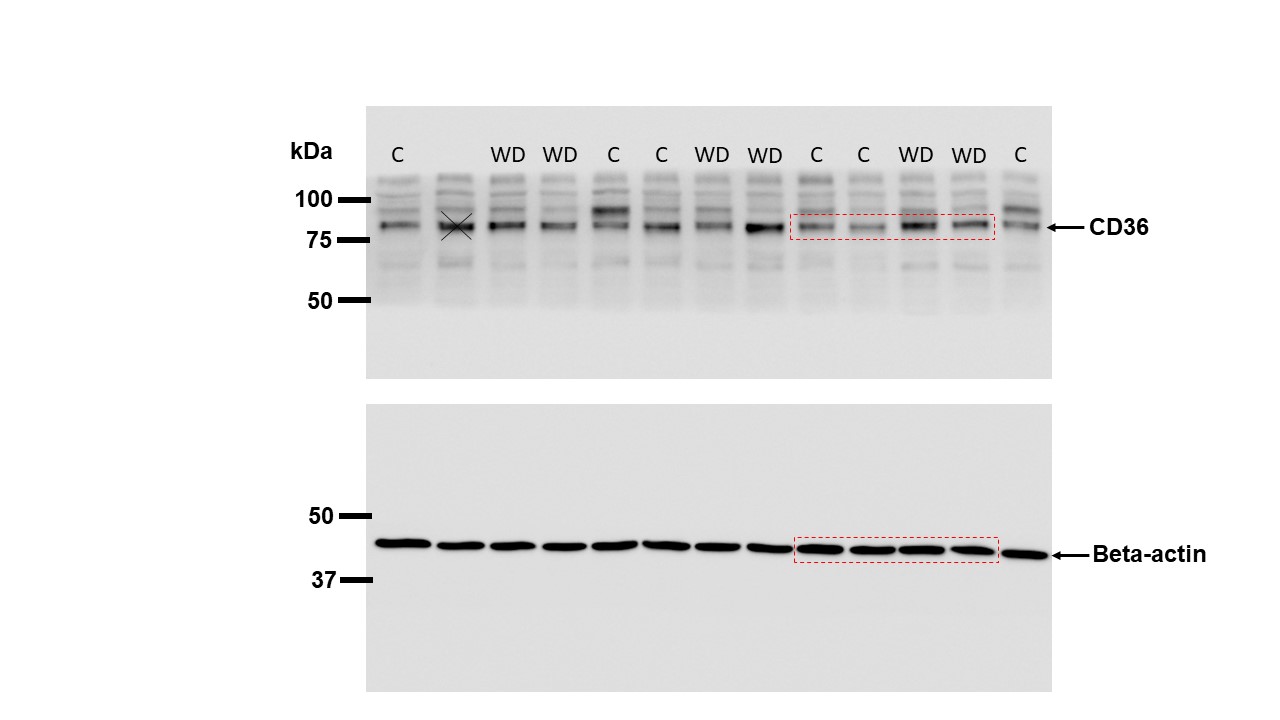
**

**

**

**

**

**
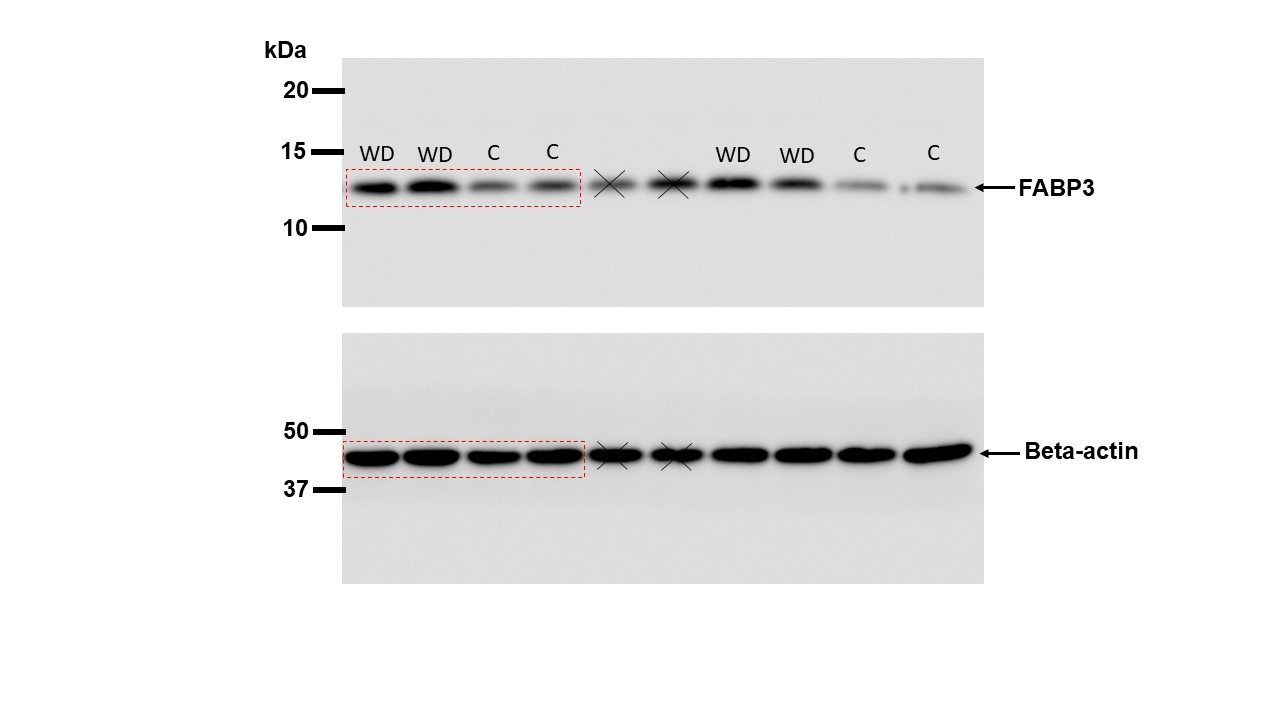
**

**

**





**Figure S2.** **The full-length blots of Figures 6A and B**. Dashed red line delimits bands shown in Figures 6A and B. The ‘X’ in the image represent bands that were not included in the analysis.
